# Avicin G is a potent sphingomyelinase inhibitor and blocks oncogenic K- and H-Ras signaling

**DOI:** 10.1038/s41598-020-65882-5

**Published:** 2020-06-04

**Authors:** Christian M. Garrido, Karen M. Henkels, Kristen M. Rehl, Hong Liang, Yong Zhou, Jordan U. Gutterman, Kwang-jin Cho

**Affiliations:** 1 Department of Biochemistry and Molecular Biology, School of Boonshoft Medical School, Wright State University, Dayton, OH 45435 United States; 2 Department of Integrative Biology and Pharmacology, McGovern Medical School, The University of Texas Health Science Center at Houston, Houston, TX 77030 United States; 3 Department of Systems Biology, The University of Texas M. D. Anderson Cancer Center, Houston, TX 77030 United States

**Keywords:** Extracellular signalling molecules, Protein translocation

## Abstract

K-Ras must interact primarily with the plasma membrane (PM) for its biological activity. Therefore, disrupting K-Ras PM interaction is a tractable approach to block oncogenic K-Ras activity. Here, we found that avicin G, a family of natural plant-derived triterpenoid saponins from *Acacia victoriae*, mislocalizes K-Ras from the PM and disrupts PM spatial organization of oncogenic K-Ras and H-Ras by depleting phosphatidylserine (PtdSer) and cholesterol contents, respectively,  at the inner PM leaflet. Avicin G also inhibits oncogenic K- and H-Ras signal output and the growth of K-Ras-addicted pancreatic and non-small cell lung cancer cells. We further identified that avicin G perturbs lysosomal activity, and disrupts cellular localization and activity of neutral and acid sphingomyelinases (SMases), resulting in elevated cellular sphingomyelin (SM) levels and altered SM distribution. Moreover, we show that neutral SMase inhibitors disrupt the PM localization of K-Ras and PtdSer and oncogenic K-Ras signaling. In sum, this study identifies avicin G as a new potent anti-Ras inhibitor, and suggests that neutral SMase can be a tractable target for developing anti-K-Ras therapeutics.

## Introduction

Ras proteins are small GTPases that primarily localize to the inner-leaflet of the plasma membrane (PM), switching between an active GTP-bound state and inactive GDP-bound state^1^. In response to epidermal growth factor stimulation or receptor tyrosine kinase activation, guanine nucleotide exchange factors activate Ras by inducing the release of the guanine nucleotides and binding of GTP^1^. Conversely, GTPase-activating proteins promote Ras GTPase activity, terminating Ras signaling by hydrolyzing GTP to GDP^1^. Activated Ras stimulates many downstream signaling pathways that control cell proliferation, differentiation and survival. Constitutively active mutant Ras are found in ~15% of all human cancers^2^. Although activating point mutations occur in all ubiquitously expressed Ras isoforms: K-, H-, and N-Ras in mammalian cells, activating point mutations in K-Ras are most prevalent, occurring in ~95% of pancreatic, ~45% colorectal, and ~35% of lung cancers^2^.

There have been increased efforts in recent years to develop strategies to directly or indirectly target cancer cells expressing oncogenic mutant K-Ras. These strategies include dysregulating cancer metabolism, blocking K-Ras downstream effectors, directly targeting oncogenic K-Ras, inhibition of synthetic lethal interactions, and perturbing K-Ras PM localization^3,4^. For the PM localization, the C-terminal CAAX motif of Ras proteins undergoes a series of posttranslational modifications to generate a cysteine farnesyl carboxy-methyl ester^5^. For H-, N- and K-Ras4A, the alternative K-Ras splicing variant, the farnesylated C-terminal cysteine in concert with palmitoylated adjacent cysteine residues anchors the protein to the PM. K-Ras4B (referred to as K-Ras herein) localizes to the PM through the farnesyl moiety and a stretch of polybasic amino acids^6–8^. While inhibitors for targeting K-Ras posttranslational modification have been unsuccessful in clinical trials, recent studies demonstrated that disrupting K-Ras PM interaction is an encouraging therapeutic strategy to block oncogenic K-Ras activity^9–14^.

Phosphatidylserine (PtdSer) is an anionic phospholipid asymmetrically localized at the inner leaflet of the PM^15^. The anionic head group of PtdSer electrostatically interacts with the polybasic domain of K-Ras, and the farnesyl-anchor of K-Ras provides high selectivity towards PtdSer over other anionic phospholipids, allowing stable K-Ras PM localization^16,17^. Consistent with the important role of PtdSer for K-Ras PM interaction, redistribution of PtdSer from the PM blocks K-Ras PM interaction, K-Ras signal output and growth of K-Ras-driven cancer cells^9,10,12,14^.

Avicins are a family of natural plant-derived triterpenoid saponins from *Acacia victoriae*, a native Australian desert tree, that possess pro-apoptotic and anti-inflammatory properties and activate anti-oxidant stress defense in mammalian cells^18–20^. Also, avicins show anti-cancer activities by lowering energy metabolism in tumors, inhibiting activation of NF-κB, and inducing tumor cell death^18,21,22^. However, the exact molecular mechanisms by which avicins prevent cancer growth and survival are not fully elucidated. In this study, we discovered that avicin G, an isomer of the avicin family mislocalizes K-Ras from the PM and disrupts PM spatial organization of oncogenic K- and H-Ras by depleting PM PtdSer and cholesterol contents, respectively. It also blocks oncogenic K- and H-Ras signal output and the growth of K-Ras-addicted pancreatic and non-small cell lung cancer cells. Avicin G also elevates lysosomal pH and inhibits the activity of acid and neutral sphingomyelinases (SMases), resulting in elevated cellular sphingomyelin (SM) levels and altered SM distribution. Furthermore, we identified neutral SMase as a tractable target for blocking oncogenic K-Ras signaling.

## Results

### Avicins translocate K-RasG12V from the plasma membrane to endomembranes

**Figure 1:**
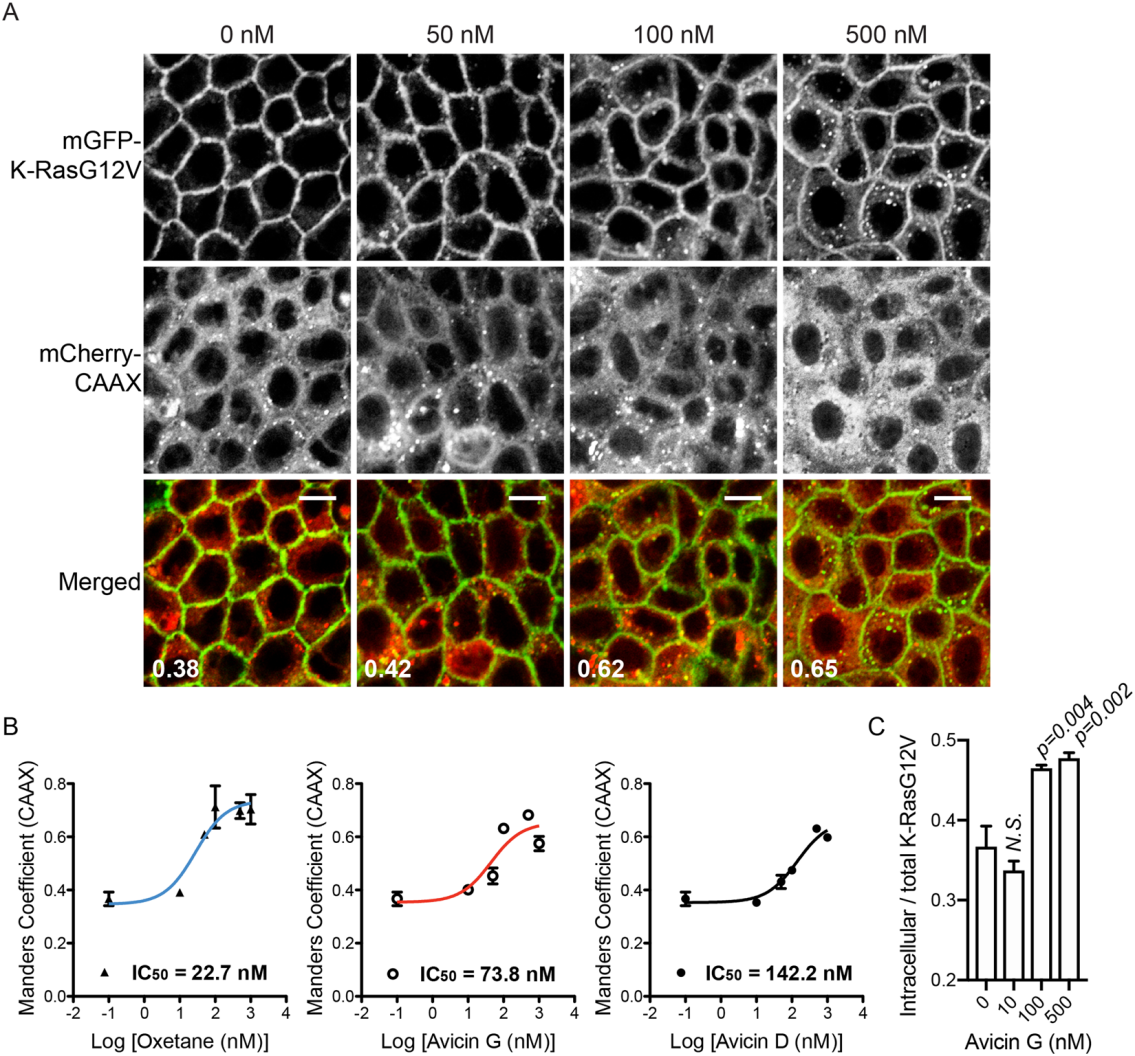
Avicin compounds mislocalize K-RasG12V from the PM. (**A**) MDCK cells stably co-expressing mGFP-K-RasG12V and mCherry-CAAX were treated with avicin G, D and oxetane for 48 h (Fig. S1). Cells were fixed with 4% PFA and imaged in a confocal microscope. Representative images of avicin G-treated cells are shown. Inserted values represent an estimated mean fraction of mCherry-CAAX co-localizing with mGFP-K-RasG12V calculated by Manders coefficient from three independent experiments. Scale bar 10 μm. (**B**) IC_50_s were estimated from the dose-response plots. (**C**) Manders coefficient values of mCherry-CAAX co-localizing with mGFP-K-RasG12V for the indicated avicin G concentrations were normalized to the total mGFP-K-RasG12V expression level measured by immunoblotting (Fig. 4A,C) to show an estimated fraction of intracellular K-RasG12V. Significant differences between control (DMSO-treated) and avicin G-treated cells were assessed using a one-way ANOVA test (*N.S*. – not significant).

A previous study reported that avicins inhibit growth of cancer cells harboring oncogenic mutant K-Ras^23^. Since K-Ras must interact primarily with the PM for its signal transduction, we analyzed the effect of avicin on K-Ras PM interaction. To test this, Madin-Darby canine kidney (MDCK) cells stably expressing monomeric green fluorescent protein (mGFP)-tagged oncogenic mutant K-Ras (K-RasG12V) and mCherry-CAAX, a generic endomembrane marker^13,24^ were treated with avicin compounds, D, G, and oxetane for 48 h. Cell images were taken using a confocal microscope, and the extent of K-RasG12V PM mislocalization was quantitated by measuring the fraction of mCherry-CAAX co-localizing with mGFP-K-RasG12V using Manders coefficient^13,25^. Our data show that all compounds mislocalized K-RasG12V from the PM with different efficacy; oxetane (IC_50_ = 22.7 nM) > avicin G (IC_50_ = 73.8 nM) > avicin D (IC_50_ = 142.2 nM) (Fig. 1A,B and S1). To further quantitate K-RasG12V PM mislocalization, intracellular K-RasG12V measured by Manders coefficient of mCherry-CAAX co-localizing with mGFP-K-RasG12V was normalized to the total mGFP-K-RasG12V expression level measured by immunoblotting (Fig. 4A,C) to calculate an estimated fraction of intracellular K-RasG12V. Our data show that the fraction of intracellular K-RasG12V increased from ~35% to ~50% in avicin G-treated cells (Fig. 1C). Taken together, our data suggest that avicins mislocalize K-RasG12V from the PM. Avicin G, like saponin, permeabilizes red blood cell PM at ~2.4 μM^26^, which is ~30-fold higher than the IC_50_ in K-Ras PM mislocalization assay. Therefore, avicin G-mediated K-RasG12V PM mislocalization is likely to be independent of its cell permeabilization activity.

**Figure 2:**
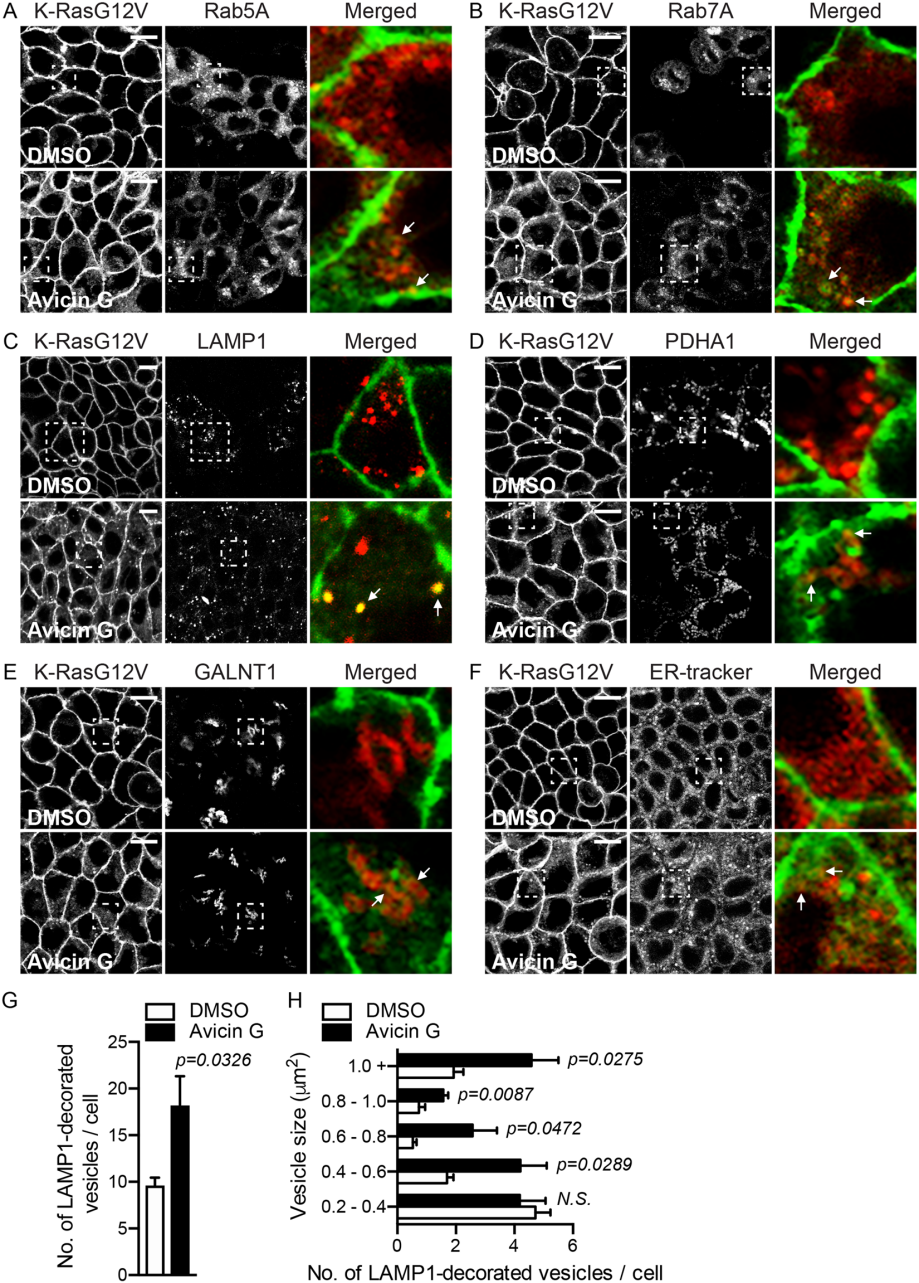
Avicin G translocates K-RasG12V from the PM to endomembranes. MDCK cells stably expressing mGFP-K-RasG12V were incubated with 500 nM avicin G for 48 in the presence of modified baculovirus encoding RFP-tagged organelle markers (**A – E**), or 2 μM ER-tracker for the final 1 h of incubation in avicin G (**F**). Cells were fixed with 4% PFA and imaged in a confocal microscope. Selected regions indicated by the white squares are shown at a higher magnification. K-RasG12V that is co-localized with the markers is indicated by arrowheads. Scale bar 10 μm. Total number of LAMP1-decorated vesicles per cell (shown in Fig. 2C) was counted (**G**), and the vesicles were categorized into their sizes (**H**). Significant differences between control (DMSO-treated) and avicin G-treated cells were assessed by Student’s *t* tests (*N.S*. – not significant).

To further characterize the K-Ras PM mislocalization, MDCK cells stably expressing mGFP-K-RasG12V only were treated with avicin G for 48 h in the presence of RFP-tagged organelle markers. Confocal microscopy data revealed that in avicin G-treated cells, K-RasG12V co-localized with Rab5A-, Rab7A-, LAMP1-, PDHA1 (pyruvate dehydrogenase E1 subunit α1)-, GALNT1 (N-acetylgalactosaminyltransferase 1)-, and ER tracker-positive structures (indicated with arrowhead in Fig. 2A–F), suggesting that K-RasG12V is translocated from the PM to early endosomes, late endosomes, lysosomes, mitochondria, the Golgi, and the ER after avicin G treatment. In addition, avicin G treatment increased the total number and size of RFP-LAMP1-decorated vesicles (Fig. 2C,G,H). Taken together with Fig. 1, our data demonstrate that avicin G translocates K-RasG12V from the PM to endomembranes.

**Figure 3:**
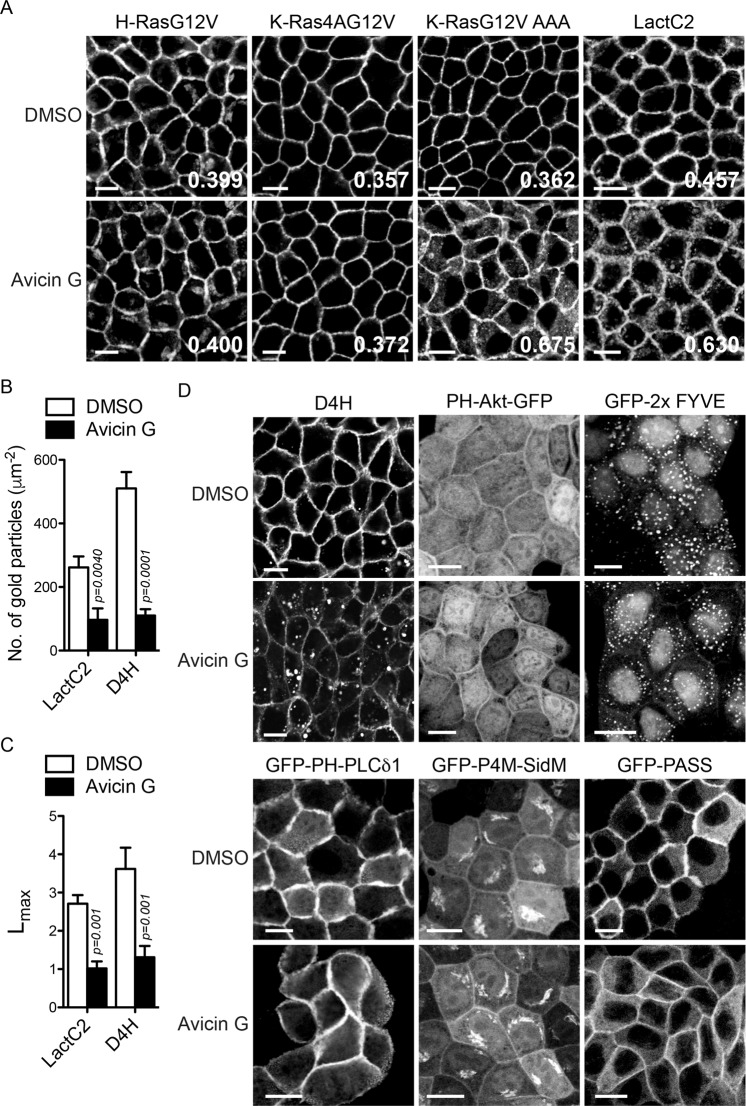
Avicin G depletes PtdSer and cholesterol from the PM. (**A**) MDCK cells stably co-expressing mCherry-CAAX and mGFP-H-RasG12V, -K-Ras4AG12V, -K-RasG12V AAA, or -LactC2 were treated with 500 nM avicin G for 48 h. Cells were fixed with 4% PFA and imaged in a confocal microscope. Inserted values represent an estimated mean fraction of mCherry-CAAX co-localizing with mGFP-tagged protein calculated by Manders coefficient from three independent experiments. Shown are representative mGFP-tagged protein images. (**B**) Apical PM sheets prepared from BHK cells expressing mGFP-LactC2 or -D4H and treated with 500 nM avicin G for 48 h were labeled with anti-GFP-conjugated gold and visualized by EM. The graph shows a mean ± S.E.M (n ≥ 15). Significant differences between control (DMSO-treated) and avicin G-treated cells were assessed by using Student’s *t* tests. (**C**) Spatial mapping of the same gold-labeled PM sheets was performed. The peak values, *L*_max_, of the respective weighted mean K-function *L(r) - r* curves are shown as bar graphs (*n* ≥ 15). Significant differences between control (DMSO-treated) and avicin G-treated cells were evaluated with bootstrap tests. (**D**) MDCK cells stably expressing mCherry-D4H or mGFP-tagged phospholipid markers were treated with 500 nM avicin G for 48 h. Cells were fixed with 4% PFA and imaged in a confocal microscope. Scale bar 10 μm.

### Avicin G depletes PtdSer and cholesterol contents at the PM

To further characterize the effects of avicin compounds on the PM localization of other Ras isoforms, MDCK cells stably co-expressing mCherry-CAAX with mGFP-H-RasG12V or -K-Ras4AG12V, the alternative K-Ras splicing variant, were treated with avicin G and imaged in a confocal microscope. Our data show that the PM localizations of H-RasG12V and K-Ras4AG12V were not disrupted by avicin G treatment, suggesting that avicin G is specific to K-Ras (Fig. 3A).

**Figure 4:**
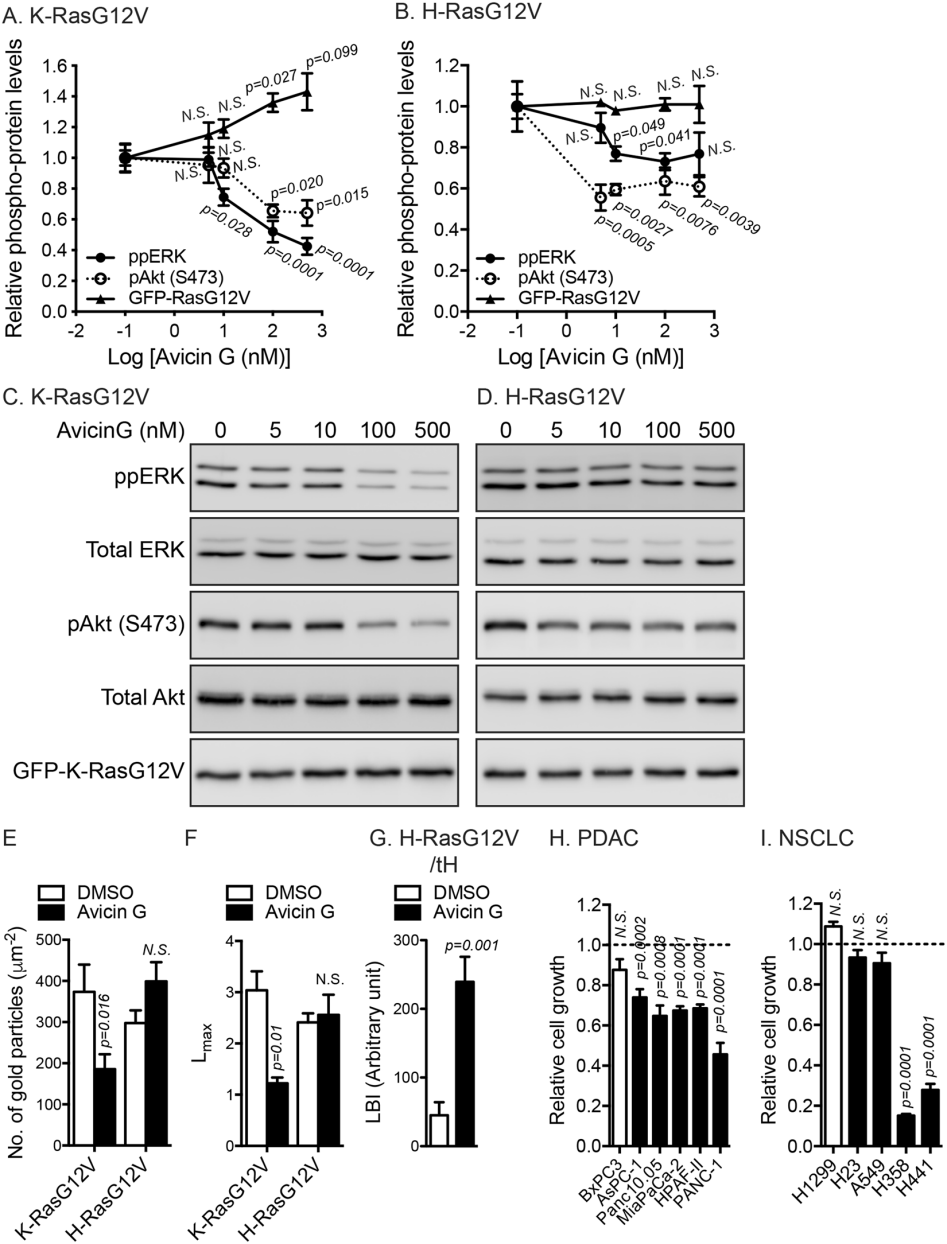
Avicin G blocks oncogenic Ras signal output and growth of K-Ras-addicted cancer cell. MDCK cells stably expressing mGFP-K-RasG12V (**A**) or -H-RasG12V (**B**) were treated with avicin G for 48 h. Cell lysates were immunoblotted for mGFP-RasG12V, phosphorylated ERK and Akt (S473). The graphs show the mean mGFP-RasG12V, phosphorylated ERK and Akt ± S.E.M from three independent experiments. Significant differences between control (DMSO-treated) and avicin G-treated cells were assessed using a one-way ANOVA test (*N.S*. – not significant). (**C,D**) Cropped representative blots are shown from three independent experiments with total ERK and Akt blots being used as loading controls. Full-length blots are shown in Fig. S3. An anti-GFP antibody was used to measure total mGFP-RasG12V levels. (**E**) Apical PM sheets prepared from BHK cells expressing mGFP-K-RasG12V or -H-RasG12V and treated with 500 nM avicin G for 48 h were labeled with anti-GFP-conjugated gold and visualized EM. The graph shows a mean ± S.E.M (n ≥ 15). Significant differences between control (DMSO-treated) and avicin G-treated cells were assessed by using Student’s *t* tests (*N.S*. - not significant). (**F**) Spatial mapping of the same gold-labeled PM sheets was performed. The peak values, *L*_max_, of the respective weighted mean K-function *L(r) - r* curves are shown as bar graphs (*n* ≥ 15). Significant differences between control (DMSO-treated) and avicin G-treated cells were evaluated with bootstrap tests (*N.S*. – not significant). (**G**) Apical PM sheets prepared from BHK cells co-expressing mGFP-H-RasG12V and mRFP-tH, a lipid raft marker, after 500 nM avicin G treatment for 48 h were labeled with anti-GFP and anti-RFP antibodies conjugated directly to 6-nm and 2-nm gold particles, respectively, and visualized by EM. The extent of co-clustering of the two proteins was analyzed by using bivariate K functions and is shown as the summary statistic LBI. The graph shows mean LBI values ± S.E.M (n ≥ 15). Significant differences between control (DMSO-treated) and avicin G-treated cells were evaluated in bootstrap tests. A panel of pancreatic ductal adenocarcinoma (PDAC) (**H**) and non-small cell lung cancer cells (NSCLC) (**I**) were plated on a 96-well plate and treated with 1.25 μM avicin G for 4 days. Complete growth medium with the drug was replaced every 24 h. Cell proliferation was analyzed using a CyQuant proliferation assay kit. The graph shows the mean cell proliferation ± S.E.M. from three independent experiments relative to that for the control cells (DMSO-treated). Open and closed bars represent cancer cells expressing WT K-Ras and oncogenic K-Ras, respectively. Significant differences between control (DMSO-treated) and avicin G-treated cells were assessed using Mann-Whitney U test (*N.S*. – not significant).

K-Ras phosphorylation at S171, S181 or T183 by protein kinase C or G dissociates K-Ras from the PM^11,27^. To test whether avicin G-mediated K-Ras PM mislocalization is through K-Ras phosphorylation, we generated MDCK cells stably expressing mCherry-CAAX and mGFP-K-RasG12V S171A, S181A and T183A (AAA) mutant, insensitive to its phosphorylation^11,27^. Cells were treated with avicin G for 48 h and imaged in a confocal microscope. Our data show that avicin G mislocalized K-RasG12V AAA mutant from the PM (Fig. 3A), suggesting that avicin G-mediated K-RasG12V PM mislocalization is independent of K-Ras phosphorylation.

K-Ras interacts with phosphatidylserine (PtdSer) at the PM via the polybasic domain and the farnesyl-anchor of K-Ras^17^, and depletion of PM PtdSer dissociates K-Ras from the PM^12,16^. To test whether avicin G mislocalizes PtdSer from the PM, we examined cellular localization of mGFP-LactC2, a well characterized PtdSer probe^12,16,28^. Our data demonstrate that avicin G redistributed LactC2 from the PM, suggesting avicin G perturbs cellular distribution of PtdSer (Fig. 3A). To directly quantify LactC2 binding to the inner PM leaflet, intact apical PM sheets from baby hamster kidney (BHK) cells expressing mGFP-LactC2 were labeled with gold-conjugated anti-GFP antibodies and analyzed by electron microscopy (EM). Our EM data reveal that avicin G treatment caused a significant decrease in immunogold labeling for mGFP-LactC2, indicating a reduction in PtdSer content at the inner leaflet of the PM (Fig. 3B and S2). A pool of PtdSer at the inner leaflet of the PM is spatially organized into nano-sized domains, which interact with the PM proteins and other lipids^12,13,29^. Further analysis of spatial organization of the remaining PtdSer at the PM reveals it was also perturbed by avicin G treatment (Fig. 3C and S2). These data suggest that avicin G attenuates the levels and spatial organization of PtdSer at the PM.

To further study the effects of avicin G on localization of other cellular lipids, MDCK cells stably expressing mGFP-tagged P4M-SidM for phosphatidylinositol (PI) 4-phosphate (P)^30^, the PH domain of Akt for PI(3,4,5)P_3_ and PI(3,4)P_2_^31,32^, 2xFYVE for PI3P^33^, PH-PLCδ1 for PI(4,5)P_2_^34^, the PASS domain of Spo20 for phosphatidic acid^35^, or mCherry-tagged D4H for cholesterol^36^ were treated with avicin G for 48 h and cell images were taken. In control cells, mCherry-D4H was predominantly localized to the PM, whereas it was internalized to vesicular structures in avicin G-treated cells (Fig. 3D). Further EM analysis of D4H probe show reduced immunogold labeling and perturbed spatial organization at the PM, suggesting avicin G abrogates the levels and spatial organization of cholesterol at the PM (Fig. 3B,C and S2). Avicin G treatment did not change the localization of other lipid markers (Fig. 3D). Taken together with Fig. 1, our data suggest that avicin G mislocalizes K-RasG12V, but not other Ras isoforms from the PM in a K-Ras phosphorylation-independent manner. It also abrogates the levels and spatial organization of PtdSer and cholesterol at the PM.

### Avicin G inhibits oncogenic Ras signal output and growth of oncogenic K-Ras-addicted cancer cells

To further study the effects of avicin G on Ras proteins, we analyzed oncogenic Ras signal output. MDCK cells stably expressing mGFP-K-RasG12V or –H-RasG12V were treated with avicin G for 48 h, and phosphorylation of ERK and Akt (S473) was measured. Our data show that avicin G significantly reduced ppERK and pAkt levels in K- and H-RasG12V cells, but the effects were greater in K-RasG12V cells (Fig. 4A–D and S3). Furthermore, avicin G treatment significantly increased the expression level of mGFP-K-RasG12V, but not -H-RasG12V (Fig. 4A–D).

Ras proteins on the PM are spatially segregated into nanodomains, called nanoclusters, that are essential for high-fidelity Ras signal transduction^37–40^. We therefore, examined the effect of avicin G on nanoclustering of oncogenic Ras on the PM. Intact apical PM sheets of BHK cells expressing mGFP-K-RasG12V or -H-RasG12V were labeled with gold-conjugated anti-GFP antibodies and analyzed by EM. Our data show a decrease in anti-GFP immunogold labeling for K-RasG12V, but not H-RasG12V after avicin G treatment, indicating loss of K-RasG12V but not H-RasG12V from the inner leaflet of the PM (Fig. 4E), consistent with our confocal microscopy data (Figs. 1–3). Spatial mapping of K- and H-RasG12V on the PM as visualized by mGFP-K-RasG12V and -H-RasG12V also reveals a significant decrease only for K-RasG12V in the peak values of the *L(r)-r* clustering statistics (Fig. 4F), indicating a reduction in the amounts of nanoclustered K-RasG12V, but not H-RasG12V that remained on the PM. Previous studies reported that lateral segregation of GTP- and GDP-loaded H-Ras fails in cells with PM cholesterol depletion, resulting in the assembly of GTP-loaded H-Ras clusters with lipid raft components that perturb MAPK activation^12,29^. We show that avicin G reduces PM cholesterol (Fig. 3B,D) and H-RasG12V signaling without disrupting H-RasG12V nanoclustering (Fig. 4B,F). Thus, we analyzed the effect of avicin G on the assembly of GTP-loaded H-Ras clusters with lipid raft by measuring the co-clustering of mGFP-H-RasG12V with a lipid raft marker, mRFP-tH (the minimal membrane-targeting domain of H-Ras). Integrated bivariate K-function (LBI) shows that mGFP-H-RasG12V and mRFP-tH were spatially segregated on the PM in control cells, whereas they were extensively co-localized after avicin G treatment, as indicated by low and high LBI values, respectively (Fig. 4G), suggesting that GTP-loaded H-Ras are mixed with GDP-loaded H-Ras proteins. Taken together, our data indicate that avicin G disrupts K-RasG12V nanoclustering, and the lateral segregation of GTP- and GDP-bound H-Ras, resulting in decreased oncogenic K- and H-Ras signal output.

To further characterize the role of avicin G on oncogenic K-Ras signaling, we performed cell proliferation assay on human pancreatic ductal adenocarcinoma (PDAC) and non-small cell lung cancer (NSCLC) cells harboring oncogenic mutant K-Ras. For control cells, we used human cancer cells expressing wild-type (WT) K-Ras (indicated by open bars). Avicin G significantly inhibited the growth of PDAC cells expressing oncogenic K-Ras (Fig. 4H). For NSCLC cells, avicin G significantly blocked the growth of H358 and H441, but not H23 and A549 cell lines (Fig. 4I). Previous studies reported that the growth of the PDAC cell lines we tested is highly dependent on oncogenic K-Ras activity, a phenomenon called K-Ras addiction^41^. For NSCLC cells, H358 and H441, but not H23 and A549 show K-Ras addiction^42^. Taken together, our data suggest that avicin G inhibits growth of K-Ras-addicted human cancer cells.

### PtdSer and cholesterol supplementation restores PM binding and spatial organization of oncogenic K- and H-Ras, respectively

**Figure 5:**
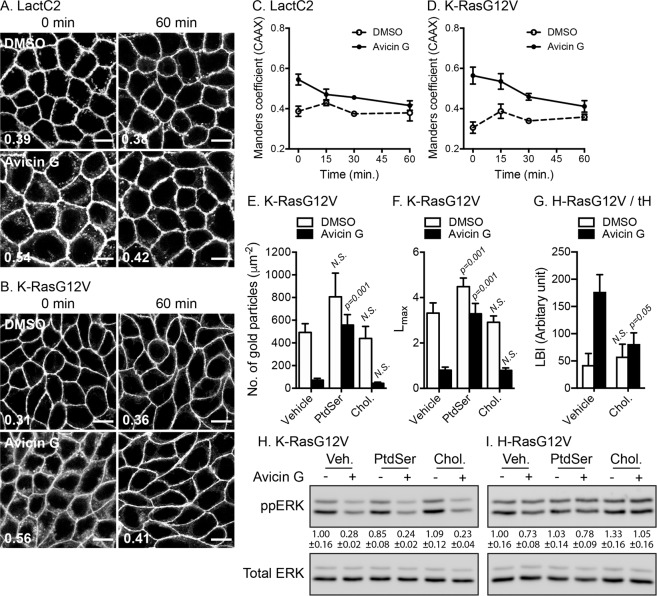
Exogenous PtdSer and cholesterol supplementation restores PM binding and nanoclustering of oncogenic K- and H-Ras, respectively. MDCK cells stably co-expressing mCherry-CAAX and mGFP-LactC2 (**A**) or -K-RasG12V (**B**) were treated with 500 nM avicin G for 48 h and further incubated with 10 μM PtdSer in the continued presence of avicin G for different time points. Cells were fixed with 4% PFA and imaged in a confocal microscope (Figs. S4 and S5). Representative images before (0 min) and 60 min after PtdSer supplementation are shown. Inserted values are an estimated mean fraction of mCherry-CAAX co-localizing with mGFP-tagged proteins calculated by Manders coefficient from three independent experiments. Scale bar 10 μm. (**C,D**) The graphs represent the PM dissociation of K-RasG12V and LactC2 quantified by Manders coefficients after PtdSer supplementation from three independent experiments. (**E**) BHK cells expressing mGFP-K-RasG12V were treated with 500 nM avicin G for 48 h and incubated with 10 μM exogenous PtdSer or cholesterol (Chol.) in the continued presence of 500 nM avicin G for another 1 h. Apical PM sheets were prepared and labeled with anti-GFP-conjugated gold and visualized EM. The graph shows a mean ± S.E.M (n ≥ 15). Significant differences between control (vehicle-treated) and lipid-supplemented cells were assessed by using a one-way ANOVA test (*N.S*. - not significant). (**F**) Spatial mapping of the same gold-labeled PM sheets was performed. The peak values, *L*_max_, of the respective weighted mean K-function *L(r) - r* curves are shown as bar graphs (*n* ≥ 15). Significant differences between control (vehicle-treated) and lipid-supplemented cells were evaluated with bootstrap tests (*N.S*. – not significant). (**G**) BHK cells co-expressing mGFP-H-RasG12V and mRFP-tH, a lipid raft marker, were treated with 500 nM avicin G treatment for 48 h and incubated with 10 μM cholesterol (Chol.) in the continued presence of 500 nM avicin G for another 1 h. Apical PM sheets were prepared, labeled with anti-GFP and anti-RFP antibodies conjugated directly to 6-nm and 2-nm gold particles, respectively, and visualized by EM. The extent of co-clustering of the two proteins was analyzed by using bivariate K functions and is shown as the summary statistic LBI. The graph shows mean LBI values ± S.E.M (n ≥ 15). Significant differences between control (vehicle-treated) and cholesterol-supplemented cells were evaluated in bootstrap tests (*N.S*. – not significant). MDCK cells stably expressing mGFP-K-RasG12V (**H**) or -H-RasG12V (**I**) were treated with 500 nM avicin G for 48 h and incubated with 10 μm exogenous PtdSer or cholesterol (Chol.) in the continued presence of 500 nM avicin G for another 1 h. Cell lysates were immunoblotted for phosphorylated ERK and quantified (means ± S.E.M from three independent experiments). Cropped representative blots are shown with total ERK blots being used as loading controls. Full-length blots are shown in Fig. S6.

To examine whether the perturbed PM binding and spatial organization of oncogenic K- and H-Ras in avicin G-treated cells are induced by depletion of PM PtdSer and cholesterol, respectively, we measured LactC2 and K-RasG12V PM association after lipid supplementation. MDCK cells stably expressing mCherry-CAAX and mGFP-LactC2 or -K-RasG12V were treated with avicin G for 48 h, followed by incubation with 10 μM PtdSer in the continued presence of avicin G. Cells were imaged at different time points and the PM association of LactC2 and K-RasG12V was quantitated using Manders coefficient. Our data show that exogenous PtdSer supplementation returned LactC2 and K-RasG12V to the PM after 15–30 min in avicin G-treated cells (Fig. 5A–D and S4–5), consistent with the time frame for the exogenous PtdSer to be displayed on the inner PM leaflet^12,13,28^. Our EM data further demonstrate that supplementation with PtdSer, but not cholesterol returned K-RasG12V to the PM and corrected K-RasG12V PM nanoclustering in avicin G-treated cells (Fig. 5E,F). However, PtdSer supplementation did not reactivate MAPK signaling in avicin G-treated K-RasG12V cells (Fig. 5H and S6). Exogenous cholesterol supplementation restored the lateral segregation of mGFP-H-RasG12V away from the lipid raft marker without altering the PM binding and nanoclustering of H-RasG12V in avicin G-treated cells (Fig. 5G and S7), and it also reactivated MAPK signaling in avicin G-treated H-RasG12V cells (Fig. 5I and S6). Taken all together, our data suggest that avicin G depletes PM cholesterol content, which perturbs H-RasG12V spatial organization, thereby inhibiting H-RasG12V/MAPK signaling.  Avicin G also perturbs the PM binding and nanoclustering of K-RasG12V through depletion of PM PtdSer. We further observed that PtdSer supplementation restored K-RasG12V nanoclustering without reactivating MAPK signaling in avicin G-treated K-RasG12V cells, suggesting that avicin G dysregulates K-Ras/Raf/MEK/ERK signal cascade at multiple levels.

### Avicin G elevates cellular sphingomyelin by inhibiting sphingomyelinase activity

**Figure 6:**
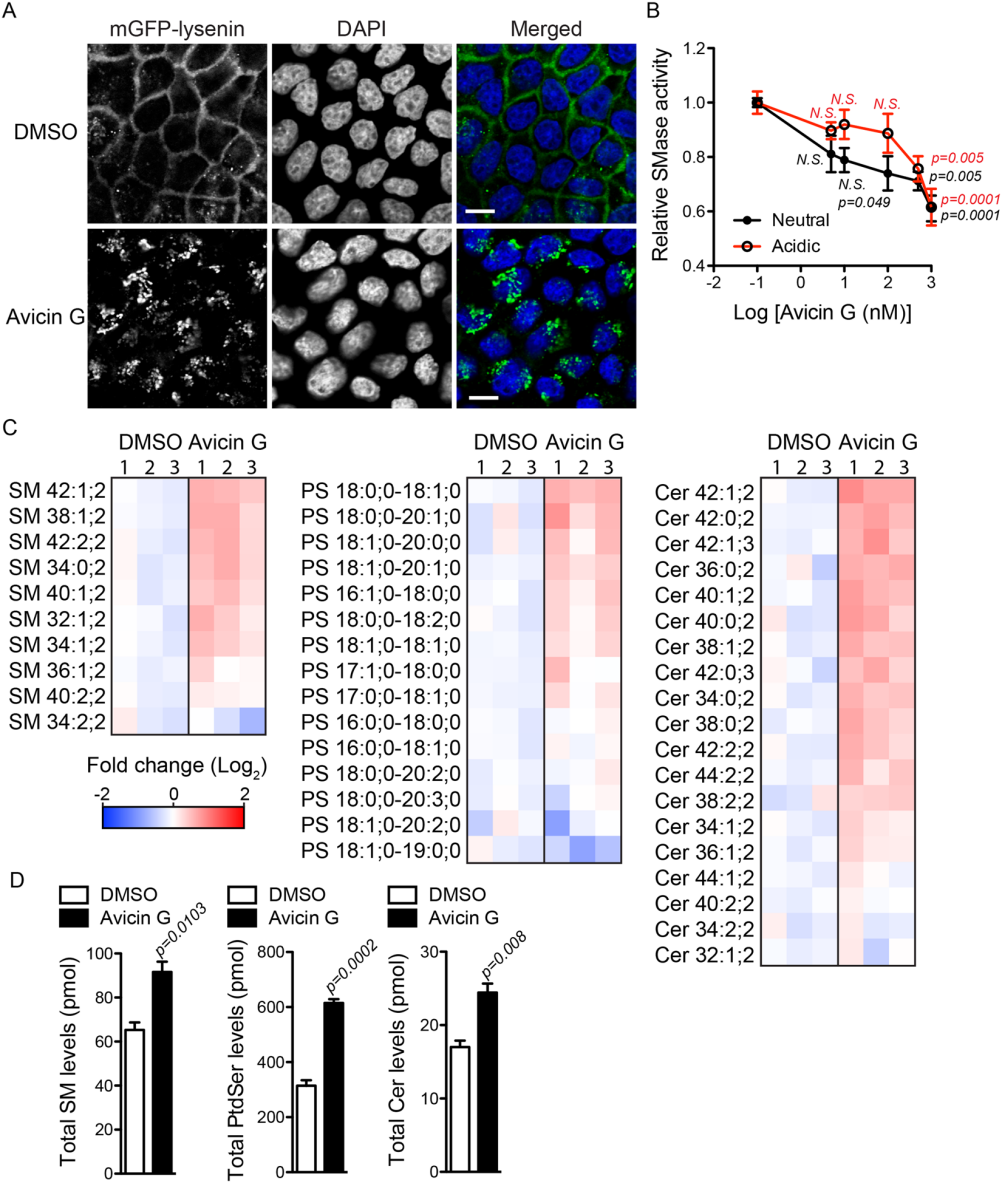
Avicin G inhibits SMases activity and perturbs SM distribution and cellular content. (**A**) WT MDCK cells treated with 500 nM avicin G for 48 h were permeabilized, co-stained with GFP-lysenin and DAPI, and imaged in a confocal microscope. Scale bar 10 μm. (**B**) Cell lysates from MDCK cells stably expressing mGFP-K-RasG12V treated with avicin G for 48 h were used to measure endogenous SMases activity. The assay was performed in an acidic (pH5.0) or a neutral (pH 7.5) environment to measure specific activities of acid SMase and neutral SMase, respectively. The graph shows the mean conversion of SM to Cer ± S.E.M from four independent experiments. Significant differences between control (DMSO-treated) and avicin G-treated cells were assessed using a one-way ANOVA test (*N.S*. – not significant). MDCK cells stably expressing mGFP-K-RasG12V were grown with or without 250 nM avicin G for 48 h, and whole cell SM, PtdSer (PS) and Cer levels were measured from three independent experiments. (**C**) A heat map was constructed to quantify the changes of different lipid species in the presence or absence of avicin G treatment. Each row represents a different species of lipid, while each column represents a single sample (sample size is n=3 for DMSO and avicin G treatment). The scaled expression values of each lipid measured is plotted in red-blue color log_2_ scale. In relation to control (DMSO-treated) cells, red and blue colored tiles indicate higher and lower lipid contents, respectively. (**D**) The graphs show the mean of total moles ± S.E.M. of indicated lipids. Significant difference between control (DMSO-treated) and avicin G-treated cells were assessed using Student’s *t* test.

Recent studies reported that perturbing cellular sphingomyelin (SM) and ceramide (Cer) levels and their distribution by dysregulating the SM biosynthesis pathway mislocalizes K-Ras and PtdSer from the PM^9,10,12,43^. To explore whether avicin G perturbs the cellular distribution of SM, WT MDCK cells were treated with avicin G for 48 h, and permeabilized and stained with recombinant GFP-lysenin, a well-studied SM probe^9,10,12,43^. Confocal microscopy shows that GFP-lysenin predominantly stained the PM in control cells, while there was a substantial accumulation of GFP-lysenin confined to intracellular vesicles in avicin G-treated cells (Fig. 6A). Next, we measured the activity of endogenous sphingomyelinase (SMase) in avicin G-treated cells. Briefly, MDCK cells stably expressing mGFP-K-RasG12V were treated with avicin G for 48 h, and cell lysates were incubated with exogenous SM to measure the conversion of SM to Cer and phosphorylcholine by endogenous SMases. Phosphorylcholine is then hydrolyzed to choline, which in turn gets oxidized generating H_2_O_2_, a readout for SM to Cer conversion^14,44^. Furthermore, since SMase converts SM to Cer in acidic or neutral cellular pH environments by SMPD1 and SMPD2/3, respectively^45–47^, we measured SMase activity in neutral (pH 7.5) and acidic (pH 5.0) environments for SMPD2/3 and SMPD1, respectively. Our data show that avicin G at >5 nM significantly inhibited conversion of SM to Cer in the neutral pH environment in a dose-dependent manner, whereas the conversion was significantly inhibited at >500 nM in the acidic environment (Fig. 6B). These data suggest that avicin G inhibits activities of neutral and acid SMases with a greater potency towards neutral SMases. A lipidomic analysis of whole cells further reveals that the level of most SM species was elevated after avicin G treatment (Fig. 6C,D), consistent with the increased SMase activity assay. Intriguingly, the lipidomic analysis also shows elevated levels of most species of PtdSer and Cer in avicin G-treated cells (Fig. 6C,D). Taken together, these data indicate that avicin G disrupts SM distribution and elevates cellular SM contents by inhibiting SMases.

### Avicin G disrupts cellular localization of SMases and lysosomal activity

**Figure 7:**
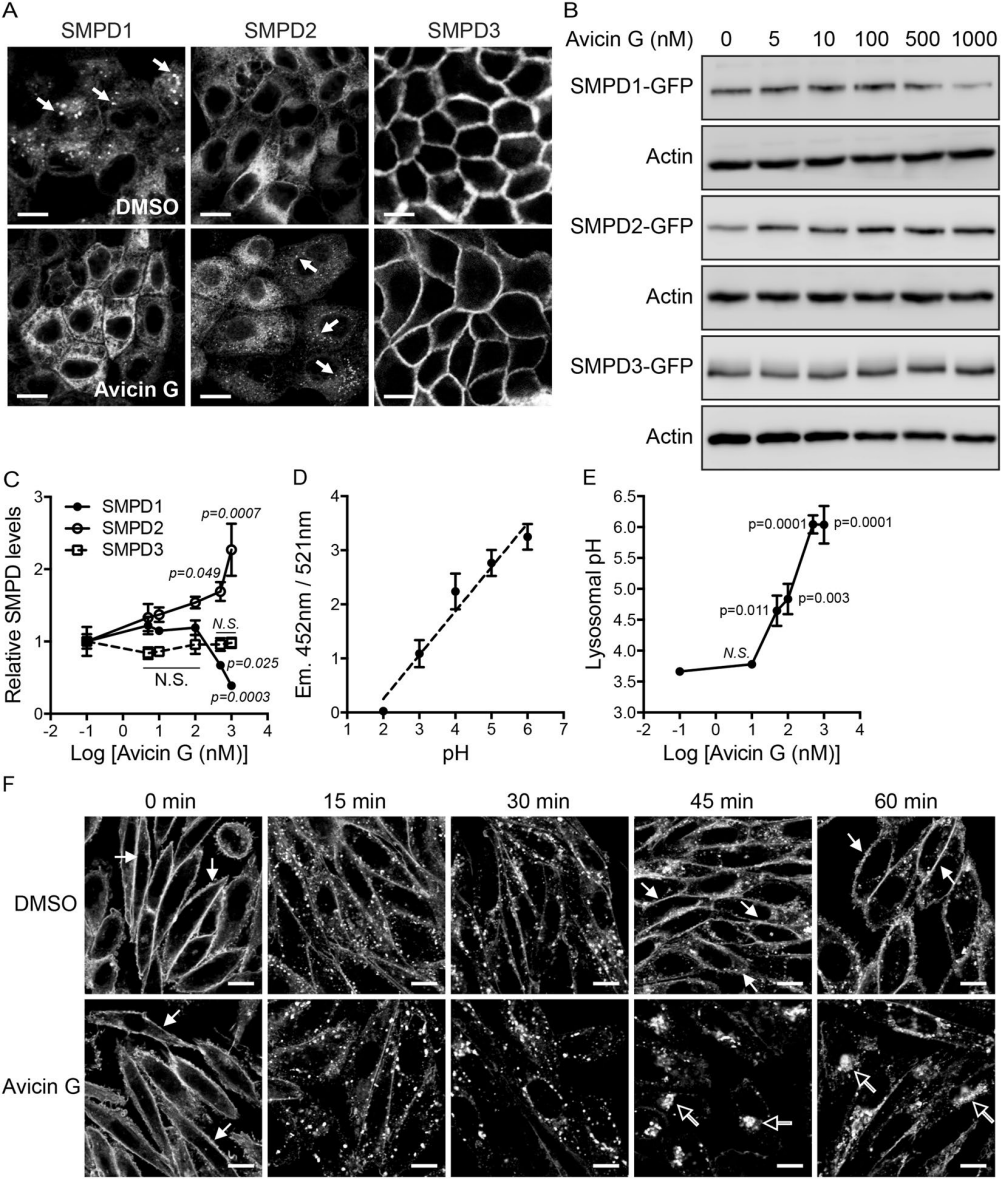
Avicin G perturbs SMases expression and localization, and EGFR recycling. (**A**) MDCK cells stably expressing SMPD1-, SMPD2-, or SMPD3-GFP were treated with 500 nM avicin G for 48 h, and cells were fixed with 4% PFA and imaged in a confocal microscope. Arrowheads indicate vesicular localization of SMPD. Scale bar 10 μm. (**B**) MDCK cells stably expressing SMPD1-, SMPD2-, or SMPD3-GFP were treated with avicin G for 48 h, and cell lysates were immunoblotted with an anti-GFP antibody to measure the total SMPD-GFP expression levels. Cropped representative blots are shown with actin blots being used as loading controls from three independent experiments. Full-length blots are shown in Fig. S8. (**C**) The graph shows the mean ± S.E.M. of SMPD expression levels from three independent experiments. Significant difference between control (DMSO-treated) and avicin G-treated cells were assessed using a one-way ANOVA test (*N.S*. – not significant). (**D**) WT MDCK cells were grown in the presence of Lysosensor Yellow/Blue dextran probe for 48 h, and equilibrated with MES calibration buffer (pH 2–6) for 15 min at room temperature. Emissions of the probe at 452 nm and 521 nm were measured by a fluorescent plate reader using a 335 nm and 381 nm excitation wavelength, respectively. The mean 452/521 nm fluorescence ratio ± S.E.M. was plotted against pH from three independent experiments, and a best fit line was plotted. (**E**) WT MDCK cells were treated with various concentrations of avicin G for 48 h in the presence of Lysosensor Yellow/Blue dextran probe. Emissions at 452 and 521 nm were measured by a fluorescent plate reader using a 335 and 381 nm excitation wavelengths, respectively. The 452/521 nm fluorescence ratio ± S.E.M. from three independent experiments were used to determine the lysosomal pH using the calibration graph from (**D**). Significant difference between control (DMSO-treated) and avicin G-treated cells were assessed using a one-way ANOVA test (*N.S*. – not significant). (**F**) CHO cells stably expressing EGFR-mGFP were treated with DMSO (vehicle) or 100 nM avicin G for 48 h. Cells were serum-starved for 2 h in the continued presence of DMSO or 100 nM avicin G, and incubated with 50 ng/ml of EGF on ice for 15 min. Excess EGF was washed away with ice-cold PBS. Cells were then incubated with fresh warm complete growth medium with DMSO or 100 nM avicin G at 37 °C and fixed at the indicated time points, followed by confocal microscopy. Representative images of DMSO- and avicin G-treated cells were shown from three independent experiments. Closed and open arrowheads indicate EGFR localized at the PM and perinuclear region, respectively. Scale bar 10 μm.

To further characterize the effect of avicin G on SMases, we examined the cellular localization and protein expression levels of SMPD1, 2, and 3 after avicin G treatment. MDCK cells stably expressing mGFP-tagged SMPD1, SMPD2 or SMPD3 were treated with avicin G for 48 h, and cells were either imaged in a confocal microscope or cell lysates were immunoblotted with an anti-GFP antibody. Our data show that lysosomal localization of SMPD1 in control cells^48^ (indicated with arrowheads) was disrupted in avicin G-treated cells (Fig. 7A). While SMPD2 was localized extensively at endomembranes in control cells^49^, avicin G treatment accumulated SMPD2 in vesicular structures (indicated with arrowheads) (Fig. 7A). Avicin G treatment did not disrupt the PM localization SMPD3^50^. Furthermore, immunoblotting shows that SMPD1-mGFP expression level was significantly reduced at >500 nM of avicin G, whereas SMPD2-mGFP level was significantly increased at >10 nM (Fig. 7B,C and S8). SMPD3-mGFP expression was not changed. (Fig. 7B,C). These data indicate that avicin G dysregulates the expression levels of SMPD1 and SMPD2 with a greater potency towards SMPD2, consistent with our SMase activity data (Fig. 7B). Taken together with SMase activity and lipidomics data in Fig. 6, our data suggest that avicin G perturbs cellular localizations and protein expressions of SMPD1 and 2, resulting in disrupted activities of SMPD1 and 2, and elevated cellular SM content.

We showed that avicin G disrupts the lysosomal localization of SMPD1 (Fig. 7A) and cholesterol distribution (Fig. 3B,D), which is regulated by Niemann-Pick C1 and 2 proteins at the lysosome^51^. Also, K-Ras expression level is elevated (Fig. 4C) and K-Ras is accumulated in the lysosome in avicin G-treated cells (Fig. 2C), where it is degraded^52^. We further showed that the total number and size of LAMP1-decorated vesicles are elevated in avicin G-treated cells (Fig. 2G,H), an indicator of lysosomal dysfunction by progressive accumulation of undigested materials^53^. Taken together, these data suggest that avicin G perturbs the lysosomal activity. To test this, we measured the lysosomal pH in avicin G-treated cells. Briefly, WT MDCK cells were treated with avicin G for 48 h in the presence of Lysosensor Yellow/Blue dextran, a fluorescent pH indicator, which has a predominantly yellow fluorescence in acidic organelles and blue fluorescence in less acidic organelles. After avicin G treatment, the ratio of 452 nm/521 nm emission was calculated, and pH values were determined from the standard curve generated by pH calibration samples (Fig. 7D). Our data show that avicin G treatment significantly increased pH values from 3.7 to 5.7 in a dose-dependent manner (Fig. 7E), suggesting avicin G elevates the lysosomal pH level. The acidic pH of lysosomal lumen provides favorable conditions for enzymatic hydrolyses, and efficient cargo sorting along recycling and degradative pathways^54,55^. Taken together with these studies, our data indicate that avicin G perturbs the lysosomal activity by elevating lysosomal pH.

### Avicin G inhibits the endocytic recycling of epidermal growth factor receptor

A recent study reported that a small synthetic molecule, G01 mislocalizes K-Ras and PtdSer from the PM, and that it also perturbs cellular distribution of SM and elevates levels of SM and Cer^9^, consistent with the effects of avicin G. Furthermore, G01 disrupts endosomal recycling epidermal growth factor receptor (EGFR) and transferrin receptor^9^. We therefore, examined whether avicin G dysregulates the endosomal recycling of EGFR. Chinese hamster ovary (CHO) cells, which have undetectable EGFR under the basal condition^56^ were generated to stably express EGFR-mGFP and treated with avicin G for 48 h. Cells were then serum-starved and pulse-labeled with EGF for 15 min, followed by confocal microscopy at the indicated time points. In control cells, EGFR-mGFP was predominantly localized to the PM at 0 min followed by rapid endocytosis at 15 min. EGFR-mGFP returned to the PM after 45 min (indicated by closed arrowheads in Fig. 7F). In avicin G-treated cells, EGFR-mGFP was endocytosed at 15 min from the PM, but it was predominantly accumulated in the perinuclear region after 45 min (indicated by open arrowheads in Fig. 7F). The endocytic recycling compartment is a series of perinuclear tubular and vesicular membranes that are often localized near the microtubule-organizing center at the perinuclear region^57^, and perturbation of recycling endosome (RE) exit of endocytosed proteins results in protein accumulation at the perinuclear region^58^. Taking our data together with these studies, it suggests that avicin G inhibits the endocytic recycling of EGFR by inhibiting the exit of EGFR from the RE.

### Neutral sphingomyelinase inhibitors disrupt K-Ras PM localization and its signaling

**Figure 8:**
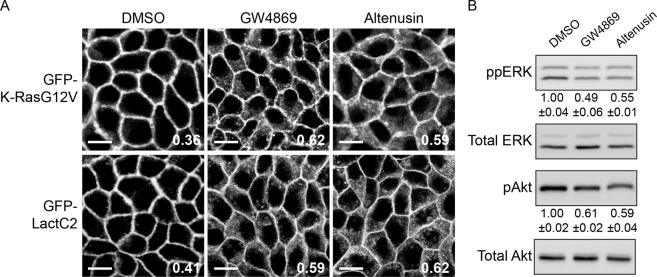
Neutral SMase inhibitors mislocalize K-Ras and PtdSer from the PM and inhibits K-Ras signal output. (**A**) MDCK cells stably co-expressing mCherry-CAAX and mGFP-K-RasG12V or -LactC2 were treated with 20 μM GW4869 or altenusin for 48 h. Cells were fixed with 4% PFA and imaged in a confocal microscope. DMSO was used as a vehicle control. Inserted values represent an estimated mean fraction of mCherry-CAAX co-localizing with mGFP-K-RasG12V or -LactC2 by Manders coefficient from three independent experiments. Shown are representative mGFP-K-RasG12V and -LactC2 images. Scale bar 10 μm. (**B)** Cell lysates of MDCK cells stably expressing mGFP-K-RasG12V treated with 20 μM GW4869 or altenusin for 48 h were immunoblotted for phosphorylated ERK and Akt (S473). Values indicate mean ppERK or pAkt ± S.E.M. from three independent experiments. Cropped representative blots are shown with total ERK and Akt blots as loading controls. Full-length blots are shown in Fig. S9.

We showed that avicin G is a potent inhibitor of neutral SMases (Fig. 6B) and inhibits oncogenic K-Ras signaling (Fig. 4). To examine whether neutral SMases can be a target for blocking oncogenic K-Ras signaling, we tested neutral SMase inhibitors for the PM localization of K-Ras and PtdSer and K-Ras signal output. MDCK cells stably co-expressing mCherry-CAAX with mGFP-K-RasG12V or -LactC2 were treated with GW4869 or altenusin for 48 h, and images were taken in a confocal microscope. Our data show that neutral SMase inhibitors mislocalized K-Ras and LactC2 from the PM (Fig. 8A). Furthermore, these inhibitors blocked phosphorylation of ERK and Akt in MDCK cells stably expressing mGFP-K-RasG12V (Fig. 8B and S9). Taken together, these data indicate that neutral SMases can be a tractable target for disrupting oncogenic K-Ras PM interaction and signal output.

## Discussion

Avicins, a family of natural plant-derived triterpenoid saponins from *Acacia victoriae* have anti-cancer activities^18,21,22^, but the exact molecular mechanism is not fully elucidated. Here, we identified that avicin G inhibits oncogenic Ras through depleting PtdSer and cholesterol contents from the inner leaflet of PM, which may contribute to the anti-cancer activities of the avicin family. Ras proteins on the PM are spatially segregated into nanoclusters that are essential for high-fidelity Ras signal transduction^38^, and PtdSer and cholesterol are important components for nanoclustering of K-Ras and H-Ras, respectively^17,29,59^. We show that avicin G depletes PM PtdSer and cholesterol contents, which  mislocalize K-Ras from the PM and perturb nanoclustering of oncogenic K- and H-Ras. This in turn, abrogates oncogenic Ras signal output. Furthermore, avicin G inhibits the growth of K-Ras-addicted PDAC and NSCLC cells in which oncogenic K-Ras activity is essential for their growth and survival^41,42^. Taken together, our data demonstrate that avicin G could be a tractable anti-Ras inhibitor.

We also show that restoring K-RasG12V PM binding and nanoclustering by PtdSer supplementation in avicin G-treated K-Ras-transformed cells do not reactivate MAPK signaling, while cholesterol supplementation restores H-RasG12V spatial organization and reactivate MAPK signaling in avicin G-treated H-Ras-transformed cells. Our study further indicates that avicin G disrupts lysosomal activity, which is important for growth of K-Ras-driven cancer cells^60–63^. Also, oncogenic mutant K-Ras stimulates macropinocytosis and autophagy, processes that require lysosomal fusion to provide an energy source^63,64^, and perturbing autophagy reduces ERK phosphorylation^65^. Taking our data together with these studies, we propose that avicin G dysregulates lysosomal activity by elevating lysosomal pH, resulting in perturbed autophagosome/lysosome fusion and thereby, abrogating ERK phosphorylation. This in turn, concomitant with the perturbed K-RasG12V PM binding and nanoclustering, inhibits oncogenic K-Ras signaling and the growth of K-Ras-addicted cancer cells.

We further demonstrate that avicin G is a potent SMase inhibitor. There are three well-studied SMases: SMPD1 (acid SMase), and SMPD 2 and 3 (neutral SMases). Avicin G inhibits the activities of acid and neutral SMases with a greater potency towards neutral SMases (Fig. 6B). One mechanism for avicin G-mediated SMPD1 inhibition is likely through perturbed lysosomal activity. It is unclear whether aberrant lysosomal activity by avicin G can account for the altered SMPD2 activity, localization and expression level.

RE is involved in maintaining K-Ras at the PM. K-Ras endocytosed from the PM is continuously sequestered from endomembranes by a chaperone protein, phosphodiesterase 6δ (PDE 6δ), and released to perinuclear membranes by an Arl2/3-dependent manner. The negative charge on the RE membranes then electrostatically traps K-Ras, from where vesicular transport returns K-Ras to the PM^66,67^. RE is enriched with cholesterol, SM and PtdSer^68,69^, and treating cells with sphingolipids blocks endosomal recycling of glucose transporter 1 and transferrin receptor^70^. Also, perturbed SM metabolism mislocalizes K-Ras and PtdSer from the PM with disrupted recycling endosomal activity^9,13,14,43^. We show that avicin G elevates cellular SM content and perturbs distribution of SM, PtdSer and cholesterol (Figs. 3 and 6) while it disrupts endosomal recycling of EGFR (Fig. 7). Taking our data together with these studies, we propose that increased cellular SM levels by avicin G alters SM content at the RE, resulting in aberrant recycling endosomal activity. This in turn, blocks PM PtdSer replenishment through RE, resulting in PtdSer depletion from the PM, which consequently mislocalizes K-Ras from the PM and abrogates K-Ras nanoclustering and its signal output. The aberrant recycling endosomal activity could further reduce PtdSer content at the RE, which disrupts the PDE6δ/RE trafficking machinery of K-Ras, further contributing to the disrupted K-Ras PM binding, nanoclustering and signaling.

Our lipidomic analysis reveals that avicin G increases cellular contents of SM, Cer and PtdSer (Fig. 6). It is unclear how SMase inhibition by avicin G elevates cellular PtdSer content. During endocytosis, PM PtdSer enters the sorting endosomes and is either recycled to the PM or delivered to lysosomes for its degradation by phospholipases^15^. Since avicin G is suggested to block lysosomal activity by altering lysosomal pH (Fig. 7), we speculate that avicin G blocks the lysosomal degradation of PtdSer, resulting in elevated PtdSer content. A previous study reported that chronic inhibition of acid SMase elevates SM and Cer^10^, consistent with our observation. We speculate that chronic inhibition of SMases may stimulate Cer production to overcome the initial Cer loss.

In sum, our study identifies avicin G as a potent acid and neutral SMase inhibitor. It also inhibits oncogenic K- and H-Ras signaling by attenuating PM PtdSer and cholesterol contents, respectively,  at the PM, suggesting that avicin G can be a tractable anti-Ras inhibitor. Our data further show that neutral SMases can be a feasible target for developing anti-K-Ras therapeutics.

## Method and Material

### Plasmids and reagents

The following antibodies used to measure Ras signaling were purchased from Cell Signaling Technology (Danvers, MA): pAkt (Ser473) (D9E) XP (Cat#4060), total Akt (pan) (40D4) (Cat#2920), p-p44/42 MAPK (Erk1/2) (Thr202/Tyr204) (D13.14.4E) XP (Cat#4370) and p44/42 MAPK (Erk1/2) (L34F12) (Cat#4696). The following antibodies used to measure housekeeping genes were purchased from Proteintech Group (Rosemont, IL): β-actin (Cat#60008-1-1 g) and GFP-tag (Cat#66002-1-lg; clone #1E10H7). cDNA of human SMPD1 (GenScript, Cat# OHu18710D), SMPD2 (GenScript, Cat# OHu30447D) and SMPD3 (Sino Biological, Cat# HG15755-G) were cloned into GFP-N1 vector. CellLight Lysosomes-RFP (Cat#C10597), Early endosomes-RFP (Cat#C10587), Late Endosomes-RFP (Cat#C10589), Golgi-RFP (Cat#10593), Mitochondria-RFP (Cat#C10601) and ER-tracker (E34250) were purchased from Invitrogen. Altenusin (Cat#SML2193) and GW4869 (Cat#567715) were purchased from MilliporeSigma. Avicin compounds were provided in collaboration with Dr. Jordan Gutterman (MD Anderson Cancer Center; Houston, TX).

### Cell culture

Madin-Darby canine kidney (MDCK) and baby hamster kidney (BHK) cells were maintained in Dulbecco’s modified eagle medium (DMEM, Cat#10569-010; Gibco). Human non-small cell lung cancer cells (NSCLCs): H1299, H23, A549, H358, and H441 were all maintained in RPMI-1640 (Cat#30-2001; ATCC). Human pancreatic ductal adenocarcinoma cells (PDACs): BxPC3, AsPC-1 and Panc 10.05 were maintained in RPMI-1640, MiaPaCa-2 and PANC-1 were maintained in DMEM and HPAF-II were maintained in EMEM (Cat#20-200; ATCC). All cancer cell lines were maintained in media supplemented with 10% FBS (Cat#16000-044; Gibco) and 2mM L-glutamine (Cat#CA009-010; GenDEPOT). Cells were routinely tested for mycoplasma (Cat#LT07-710; MycoAlert PLUS Mycoplasma Detection Kit; Lonza; Rockland, ME). All cell lines were maintained in 37 °C incubator with 5% CO_2_ injection.

### Western blotting

Preparation of cell lysates and immunoblotting were performed as described previously^71^. Briefly, cells were washed twice with ice-cold 1x phosphate-buffered saline (PBS). Cells were harvested in lysis buffer B containing 50 mM Tris-Cl (pH 7.5), 75 mM NaCl, 25 mM NaF, 5 mM MgCl_2_, 5 mM EGTA, 1 mM DTT, 100 μM NaVO_4_, 1% NP-40 plus protease and phosphatase inhibitors. SDS-PAGE and immunoblotting were generally performed using 20–25 μg of lysate from each sample group. Signals were detected by enhanced chemiluminescence (Cat# 34578 and 34075, Thermo Fisher Scientific) and imaged using an Amersham Imager 600 (GE Healthcare). ImageJ software (v1.52) was used to quantitate band intensity.

### Lysenin staining

A maltose binding protein (MBP)–GFP–lysenin fragment (amino acid residues 161 to 297) was purified as previously described^43^. WT MDCK cells were fixed with 4% PFA for 30 min, and permeabilized with 0.05% saponin for 30 min at room temperature (RT), followed by labeling with (15 μg/mL) MBP-GFP-lysenin for 15 min at RT. Cells were then quenched with NH_4_Cl (50 mM) in dark for 10 min, and further labeled with DAPI (2.5 μg/mL) in dark for 10 min. Cells were imaged using a confocal microscope.

### Counting LAMP1-decorated vesicles

Counting the total number of LAMP1-decorated vesicles and categorizing them by their sizes were performed using *Analyze Particles* function in ImageJ software (v1.52).

### Proliferation assay

Non-small cell lung cancer cells (H1299, H23, A549, H358, H441) and pancreatic ductal adenocarcinoma cells (BxPC3, AsPC-1, Panc10.05, MiaPaCa-2, HPAF-II, PANC-1) were plated at 1 ×10^4^ onto a 96-well plate. Next day, cells were incubated with 1250 nM avicin G or DMSO (control) in 100 μL complete growth media for 3 days. Fresh complete growth medium with the compound were replaced every 24 h. Cell proliferation was assayed using CyQuant NF Cell Proliferation Assay Kit (Invitrogen, Cat#C35006) according to the manufacturer’s instructions. Plates were read using BioTek Synergy H1 microplate reader (excitation λ = 480 nm, emission λ = 530 nm).

### Lipidomic analysis

MDCK cells stably expressing mGFP-K-RasG12V were treated with 250 nM avicin G or DMSO for 48 h. Cells were collected in Dulbecco’s PBS (DPBS, Cat# 14190144, Invitrogen) and 4.5 ×10^5^ cells in 300 μL DPBS were prepared for lipidomics. Lipid extraction and analysis using electron spray ionization and MS/MS were performed at Lipotype GmbH (Dresden, Germany), as described previously^72,73^. Automated processing of acquired mass spectra, identification, and quantification of detected lipid species were done by LipidXplorer software. Only lipid identifications with a signal-to-noise ratio >5, an absolute abundance of at least 1 pmol, and a signal intensity 5-fold higher than in corresponding blank samples were considered for further data analysis. The abundance of lipids is presented as a heap map with log_2_ scale, relative to control (DMSO-treated) cells, and as pmol of lipids/cell.

### Sphingomyelinase activity assay

The SMase activity assay was performed with Amplex Red SMase Assay Kit (A12220, Invitrogen) as described previously^14^. Briefly for the neutral SMase assay, 10 μg of whole-cell lysates (at 1 μg/μl concentration) prepared in lysis buffer B without DTT was mixed with 40 μl of 1x reaction buffer (0.5 M Tris-HCl pH 7.4, 50 mM MgCl_2_), and loaded on a well of black 96-well plate. 50 μl of Amplex Red reaction mixture (100 μM Amplex Red reagent, 2 U/ml horseradish peroxidase, 0.2 U/ml choline oxidase, 8 U/ml alkaline phosphatase, and 0.5 mM sphingomyelin in 1x reaction buffer) was added to each well, and the plate was incubated in dark at 37 °C for 60 min. The fluorescence was measured using BioTek Synergy H1 microplate reader (excitation λ = 540 nm, emission λ = 590 nm). 0.1 U/ml SMase and 1x reaction buffer were used as a positive and a negative control, respectively. For the acid SMase assay, 10 μg of whole-cell lysates (at 1 μg/μl concentration) prepared in lysis buffer B without DTT was mixed with 40 μl of low-pH buffer (50 mM sodium acetate, pH 5.0), and loaded on a well of black 96- well plate. 5 μl of 5 mM sphingomyelin was added to each well, and the plate was incubated in dark in 37 °C for 1 h. After the incubation, the pH was raised to 7.4 by adding 50 μl of Amplex Red reaction mixture [100 μM Amplex Red reagent, 2 U/ml horseradish peroxidase, 0.2 U/ml choline oxidase, 8 U/ml alkaline phosphatase in high-pH buffer (100 mM Tris-HCl, pH 8.0)]. The plate was further incubated in the dark in 37 °C for 60 min, and the fluorescence was measured using BioTek Synergy H1 microplate reader (excitation λ = 540 nm, emission λ = 590 nm). 0.1 U/ml SMase and low-pH buffer were used as a positive and negative control, respectively.

### Measuring lysosomal pH

For a calibration curve, 1.56 ×10^4^ WT MDCK cells were plated onto a 96-well plate. Next day, cells were incubated with 1% vehicle (DMSO) and 100 μg Lysosensor Yellow/Blue dextran (25 μg/μL) (Cat#L22460, Invitrogen) in 100 μL complete growth medium. 48 h later, cells were washed twice with 1x PBS, and incubated with the following MES ((2-(N-morpholino)ethanesulfonic acid) pH calibration curve buffers (125 mM KCl, 25 mM NaCl, 10 μM monensin and 25 mM MES) for 15 min at RT^74^. pH was adjusted to the appropriate final pH (pH 2–6) using 1 N NaOH or 1 N HCl. The fluorescence was measured using BioTeck Synergy H1 microplate reader (ex. λ = 355 nm and em. λ = 452 nm for CFP, and ex. λ = 381 nm and em. λ = 521 nm for YFP). The ratio of 452 nm / 521 nm was calculated and plotted against pH, and a best fit line was plotted using Microsoft Excel software.

For measuring lysosomal pH, 1.56 ×10^4^ WT MDCK cells were plated onto a 96-well plate. Next day, cells were incubated with 1% vehicle (DMSO) or various concentrations of avicin G with 100 μg Lysosensor Yellow/Blue dextran (25 μg/μL) in 100 μL complete growth medium. 48 h later, cells were washed twice with 1x PBS, and the fluorescence was measured using BioTeck Synergy H1 microplate reader (ex. λ = 355 nm and em. λ = 452 nm for CFP, and ex. λ = 381 nm and em. λ = 521 nm for YFP). The ratio of 452 nm / 521 nm were used to determine the lysosomal pH using the calibration graph.

### Lipid supplementation

Lipids were prepared and supplemented to cells as described previously^12,28^. Briefly, brain phosphatidylserine (PtdSer; 830032 C) and cholesterol (Chol; 700000, both from Avanti Polar Lipids) were dried under a vacuum in a glass vial to remove the solvent, reconstituted in 2 mL serum-free growth medium using a bath sonicator, and diluted with complete growth medium to a final concentration of 10 μM. Cells were washed twice with 1x PBS and further incubated with the lipid for indicated time points at 37 °C in 5% CO_2_.

### Electron microscopy (EM)-spatial analysis

#### EM-univariate spatial analysis

The univariate nanoclustering analysis quantifies the extent of spatial segregation of a single species of immunogold-labeling on intact PM sheets^17,75,76^. Following DMSO vehicle control or 500 nM Avicin G treatment for 48 hours, Intact apical PM sheets of BHK cells expressing a GFP-tagged RasG12V mutant, -LactC2 or -D4H were attached to copper EM grids. The PM sheets were fixed with 4% paraformaldehyde (PFA) and 0.1% glutaraldehyde, then immunolabeled with 4.5-nm gold nanoparticles coupled with anti-GFP antibody and coated with uranyl acetate for negative-staining. Transmission EM (TEM) at 100,000X magnification was used to image gold particles on the PM sheets. ImageJ assigned the coordinates of every gold particle on a selected 1μm^2^ area on intact PM sheet. Spatial distribution of the gold particles was calculated the Ripley’s K-function. The null hypothesis of the K-function analysis is that gold particles in the selected area are randomly distributed (Equations 1 and 2):^77,78^

1 $$K(r)={\rm{A}}{n}^{-2}\sum _{i\ne j}{w}_{ij}1({x}_{i}-{x}_{j}\le r)$$

2 $$L(r)-r=\sqrt{\frac{K(r)}{\pi }}-r$$

where *K(r)* designates the univariate (single population) K-function for *n* gold particles in an area of *A*; *r* is a length scale (1 → 240 nm with an increment of 1 nm); ||. || is Euclidean distance, for which 1(^.^) = 1 if ||*x*_*i*_-*x*_*j*_|| ≤ r and 1(^.^) = 0 if ||*x*_*i*_-*x*_*j*_|| > r. To correct a potential edge effect along the edges of the EM images, we draw a circle with the center at *x*_*i*_ and radius ||*x*_*i*_-*x*_*j*_||. The term *w*_*ij*_^−1^ defines the fraction of the circumference of the circle. *L*(*r*) – *r* is a linear transformation of *K*(*r*), and is normalized against the 99% confidence interval (99% C.I.) estimated from Monte Carlo simulations. A perfectly random distribution for all gold labeling is indicated by a *L*(*r*) - *r* value of 0, while statistically significant nanoclustering is denoted by a *L*(*r*) - *r* value above the 99% C.I. of 1. For each condition, ≥ 15 PM sheets were analyzed and pooled, with the peak value on the *L*(*r*) - *r* function curve termed as *L*_*max*_ to summarize optimal nanoclustering. To evaluate the potential statistical significance between the DMSO vehicle control and the Avicin G treatment for each gold-labeled construct, our bootstrap tests compare experimental point patterns with 1000 bootstrap samples^17,75^.

### EM-Bivariate co-localization analysis

To quantify a potential co-clustering between GFP-tagged and RFP-tagged proteins / peptides, we used the EM bivariate K-function co-localization analysis^17,75,76^. BHK cells co-expressing GFP-H-RasG12V and RFP-tH were treated with either DMSO vehicle control or 500 nM Avicin G for 48 hours. Intact apical PM sheets of these cells were attached to EM grids, fixed and then immunolabeled with 2-nm gold linked to anti-RFP antibody and 6 nm gold conjugated to anti-GFP antibody, respectively. ImageJ assigned X / Y coordinates of each gold nanoparticle, which were used in a bivariate K-function to calculate the co-localization between 2-nm and 6-nm gold populations. The null hypothesis for this analysis is that the two gold populations spatially segregate from each other. (Eqs. 3-5):^78^

3 $${K}_{biv}(r)={({n}_{b}+{n}_{s})}^{-1}[{n}_{b}{K}_{sb}(r)+{n}_{s}{K}_{bs}(r)]$$

4 $${K}_{bs}(r)=\frac{A}{{n}_{b}{n}_{s}}\mathop{\sum }\limits_{i=1}^{{n}_{b}}\mathop{\sum }\limits_{j=1}^{{n}_{s}}{w}_{ij}1({x}_{i}-{x}_{j}\le r)$$

5 $${K}_{sb}(r)=\frac{A}{{n}_{b}{n}_{s}}\mathop{\sum }\limits_{i=1}^{{n}_{s}}\mathop{\sum }\limits_{j=1}^{{n}_{b}}{w}_{ij}1({x}_{i}-{x}_{j}\le r)$$

6 $${L}_{biv}(r)-r=\sqrt{\frac{{K}_{biv}(r)}{\pi }}-r$$

where *K*_*biv*_(*r*) denotes the bivariate estimator containing 2 individual bivariate K-functions: *K*_*bs*_(*r*) defines the spatial distribution of all the big gold particles (*b* = 6-nm big gold) with respect to each small gold particle (*s* = 2-nm small gold); and *K*_*sb*_(*r*) depicts the spatial distribution of all the small gold with respect to each big gold. The value of *n*_*b*_ is the number of big gold particles and *n*_*s*_ is the number of small gold in a PM area of *A*. Other parameters follow the same denotation as described in the univariate K-function analysis (Equations 1 and 2). *L*_*biv*_(*r*)-*r* is a linear transformation of *K*_*biv*_(*r*), and is then normalized against the 95% confidence interval (95% C.I.). An *L*_*biv*_(*r*)-*r* value of 0 denotes spatial separation between 2-nm/6-nm gold particles, while an *L*_*biv*_(*r*)-*r* value above the 95% C.I. of 1 indicates statistically significant co-localization. Area under each *L*_*biv*_(*r*)-*r* curve within a fixed range 10 < *r* < 110 nm was then integrated to yield a parameter of bivariate *L*_*biv*_(*r*)-*r* integrated (or *LBI*):

7 $$\,LBI={\int }_{10}^{110}Std\,{L}_{biv}(r)-r.\,dr\,$$

For each DMSO/Avicin treatment, at least 15 apical PM sheets were analyzed and pooled. Statistical significance between DMSO vehicle and Avicin treatment was gauged by comparing experimental point patterns against 1000 bootstrap samples as described above^17,75^.

### Confocal microscopy

Cells were grown on coverslips and fixed with 4% paraformaldehyde, followed by 50 mM NH_4_Cl treatment, and imaged by confocal microscopy (Olympus FV1000) using a 60x objective.

### Statistical analysis

Prism (v8.0, GraphPad Software) was used for one-way ANOVA tests, Student’s *t* tests and Mann-Whitney U tests.

## Supplementary information

Supplementary Information.

## References

[1] Baines AT, Xu D, Der CJ (2011). Inhibition of Ras for cancer treatment: the search continues. Future Med Chem.

[2] Prior, I. A., Lewis, P. D. & Mattos, C. A comprehensive survey of Ras mutations in cancer. *Cancer Res* **72**, 2457-2467, doi:10.1158/0008-5472.CAN-11-261272/10/2457 [pii] (2012). 10.1158/0008-5472.CAN-11-2612 PMC3354961 22589270

[3] Papke B, Der CJ (2017). Drugging RAS: Know the enemy. Science.

[4] Gorfe, A. A. & Cho, K. J. Approaches to inhibiting oncogenic K-Ras. *Small GTPases.* **2019**, 1–10, 10.1080/21541248.2019.1655883 (2019). 10.1080/21541248.2019.1655883 PMC7849769 31438765

[5] Hancock JF (2003). Ras proteins: different signals from different locations. Nat Rev Mol Cell Biol.

[6] Hancock, J. F., Paterson, H. & Marshall, C. J. A polybasic domain or palmitoylation is required in addition to the CAAX motif to localize p21ras to the plasma membrane. *Cell* 63, 133-139, doi:0092-8674(90)90294-O [pii] (1990). 10.1016/0092-8674(90)90294-o 2208277

[7] Hancock JF, Magee AI, Childs JE, Marshall CJ (1989). All ras proteins are polyisoprenylated but only some are palmitoylated. Cell.

[8] Gutierrez L, Magee AI, Marshall CJ, Hancock JF (1989). Post-translational processing of p21ras is two-step and involves carboxyl-methylation and carboxy-terminal proteolysis. EMBO J.

[9] Tan L, Cho KJ, Neupane P, Capon RJ, Hancock JF (2018). An oxanthroquinone derivative that disrupts RAS plasma membrane localization inhibits cancer cell growth. J Biol Chem.

[10] van der Hoeven, D. *et al*. Sphingomyelin Metabolism Is a Regulator of K-Ras Function. *Mol Cell Biol* **38**, 10.1128/MCB.00373-17 (2018). 10.1128/MCB.00373-17 PMC5770534 29158292

[11] Cho KJ (2016). AMPK and Endothelial Nitric Oxide Synthase Signaling Regulates K-Ras Plasma Membrane Interactions via Cyclic GMP-Dependent Protein Kinase 2. Mol Cell Biol.

[12] Cho KJ (2016). Inhibition of Acid Sphingomyelinase Depletes Cellular Phosphatidylserine and Mislocalizes K-Ras from the Plasma Membrane. Mol Cell Biol.

[13] Cho, K. J. *et al*. Staurosporines disrupt phosphatidylserine trafficking and mislocalize Ras proteins. *J Biol Chem* **287**, 43573-43584, doi:10.1074/jbc.M112.424457M112.424457 [pii] (2012). 10.1074/jbc.M112.424457 PMC3527944 23124205

[14] Tan Lingxiao, Cho Kwang-Jin, Kattan Walaa E., Garrido Christian M., Zhou Yong, Neupane Pratik, Capon Robert J., Hancock John F. (2019). Acylpeptide hydrolase is a novel regulator of KRAS plasma membrane localization and function. Journal of Cell Science.

[15] Leventis PA, Grinstein S (2010). The distribution and function of phosphatidylserine in cellular membranes. Annu Rev Biophys.

[16] Yeung T., Gilbert G. E., Shi J., Silvius J., Kapus A., Grinstein S. (2008). Membrane Phosphatidylserine Regulates Surface Charge and Protein Localization. Science.

[17] Zhou Y (2017). Lipid-Sorting Specificity Encoded in K-Ras Membrane Anchor Regulates Signal Output. Cell.

[18] Wang H, Haridas V, Gutterman JU, Xu ZX (2010). Natural triterpenoid avicins selectively induce tumor cell death. Commun Integr Biol.

[19] Gutterman JU (2005). Effects of the tumor inhibitory triterpenoid avicin G on cell integrity, cytokinesis, and protein ubiquitination in fission yeast. Proc Natl Acad Sci USA.

[20] Haridas V (2001). Avicins: triterpenoid saponins from Acacia victoriae (Bentham) induce apoptosis by mitochondrial perturbation. Proc Natl Acad Sci USA.

[21] Haridas V, Arntzen CJ, Gutterman JU (2001). Avicins, a family of triterpenoid saponins from Acacia victoriae (Bentham), inhibit activation of nuclear factor-kappaB by inhibiting both its nuclear localization and ability to bind DNA. Proc Natl Acad Sci USA.

[22] Haridas V (2007). Avicins, a novel plant-derived metabolite lowers energy metabolism in tumor cells by targeting the outer mitochondrial membrane. Mitochondrion.

[23] Mujoo K (2001). Triterpenoid saponins from Acacia victoriae (Bentham) decrease tumor cell proliferation and induce apoptosis. Cancer Res.

[24] Choy E (1999). Endomembrane trafficking of ras: the CAAX motif targets proteins to the ER and Golgi. Cell.

[25] Manders EMM, Verbeek FJ, Aten JA (1993). Measurement of co-localization of object in dual-colour confocal images. Journal of Microscopy.

[26] Arias M, Quijano JC, Haridas V, Gutterman JU, Lemeshko VV (2010). Red blood cell permeabilization by hypotonic treatments, saponin, and anticancer avicins. Biochim Biophys Acta.

[27] Bivona TG (2006). PKC regulates a farnesyl-electrostatic switch on K-Ras that promotes its association with Bcl-XL on mitochondria and induces apoptosis. Mol Cell.

[28] Miller Taylor E., Henkels Karen M., Huddleston Mary, Salisbury Richard, Hussain Saber M., Sasaki Atsuo T., Cho Kwang-Jin (2019). Depletion of phosphatidylinositol 4-phosphate at the Golgi translocates K-Ras to mitochondria. Journal of Cell Science.

[29] Zhou Yong, Liang Hong, Rodkey Travis, Ariotti Nicholas, Parton Robert G., Hancock John F. (2013). Signal Integration by Lipid-Mediated Spatial Cross Talk between Ras Nanoclusters. Molecular and Cellular Biology.

[30] Hammond GR, Machner MP, Balla T (2014). A novel probe for phosphatidylinositol 4-phosphate reveals multiple pools beyond the Golgi. J Cell Biol.

[31] Franke TF, Kaplan DR, Cantley LC, Toker A (1997). Direct regulation of the Akt proto-oncogene product by phosphatidylinositol-3,4-bisphosphate. Science.

[32] James SR (1996). Specific binding of the Akt-1 protein kinase to phosphatidylinositol 3,4,5-trisphosphate without subsequent activation. Biochem J.

[33] Stenmark H, Aasland R, Toh BH, D’Arrigo A (1996). Endosomal localization of the autoantigen EEA1 is mediated by a zinc-binding FYVE finger. J Biol Chem.

[34] Garcia P (1995). The pleckstrin homology domain of phospholipase C-delta 1 binds with high affinity to phosphatidylinositol 4,5-bisphosphate in bilayer membranes. Biochemistry.

[35] Zhang F (2014). Temporal production of the signaling lipid phosphatidic acid by phospholipase D2 determines the output of extracellular signal-regulated kinase signaling in cancer cells. Mol Cell Biol.

[36] Maekawa, M. & Fairn, G. D. Complementary probes reveal that phosphatidylserine is required for the proper transbilayer distribution of cholesterol. *J Cell Sci* **128**, 1422-1433, 10.1242/jcs.164715jcs.164715 [pii] (2015). 10.1242/jcs.164715 25663704

[37] Cho, K. J. & Hancock, J. F. Ras nanoclusters: a new drug target? *Small GTPases* **4**, 57-60, 10.4161/sgtp.2314523145 [pii] (2013). 10.4161/sgtp.23145 PMC3620104 23419283

[38] Tian Tianhai, Harding Angus, Inder Kerry, Plowman Sarah, Parton Robert G., Hancock John F. (2007). Plasma membrane nanoswitches generate high-fidelity Ras signal transduction. Nature Cell Biology.

[39] Prior IA, Muncke C, Parton RG, Hancock JF (2003). Direct visualization of Ras proteins in spatially distinct cell surface microdomains. J Cell Biol.

[40] Cho Kwang-jin, Kasai Rinshi S., Park Jin-Hee, Chigurupati Sravanthi, Heidorn Sonja J., van der Hoeven Dharini, Plowman Sarah J., Kusumi Akihiro, Marais Richard, Hancock John F. (2012). Raf Inhibitors Target Ras Spatiotemporal Dynamics. Current Biology.

[41] Hayes TK (2016). Long-Term ERK Inhibition in KRAS-Mutant Pancreatic Cancer Is Associated with MYC Degradation and Senescence-like Growth Suppression. Cancer Cell.

[42] Singh A (2009). A gene expression signature associated with “K-Ras addiction” reveals regulators of EMT and tumor cell survival. Cancer Cell.

[43] Maekawa M, Lee M, Wei K, Ridgway ND, Fairn GD (2016). Staurosporines decrease ORMDL proteins and enhance sphingomyelin synthesis resulting in depletion of plasmalemmal phosphatidylserine. Sci Rep.

[44] Petrache I (2005). Ceramide upregulation causes pulmonary cell apoptosis and emphysema-like disease in mice. Nat Med.

[45] Callahan JW, Jones CS, Davidson DJ, Shankaran P (1983). The active site of lysosomal sphingomyelinase: evidence for the involvement of hydrophobic and ionic groups. J Neurosci Res.

[46] Liu B, Hassler DF, Smith GK, Weaver K, Hannun YA (1998). Purification and characterization of a membrane bound neutral pH optimum magnesium-dependent and phosphatidylserine-stimulated sphingomyelinase from rat brain. J Biol Chem.

[47] Marchesini N, Hannun YA (2004). Acid and neutral sphingomyelinases: roles and mechanisms of regulation. Biochem Cell Biol.

[48] Lee CY (2007). Carboxyl-terminal disulfide bond of acid sphingomyelinase is critical for its secretion and enzymatic function. Biochemistry.

[49] Fensome AC, Rodrigues-Lima F, Josephs M, Paterson HF, Katan M (2000). A neutral magnesium-dependent sphingomyelinase isoform associated with intracellular membranes and reversibly inhibited by reactive oxygen species. J Biol Chem.

[50] Hinkovska-Galcheva V (1998). Activation of a plasma membrane-associated neutral sphingomyelinase and concomitant ceramide accumulation during IgG-dependent phagocytosis in human polymorphonuclear leukocytes. Blood.

[51] Sleat DE (2004). Genetic evidence for nonredundant functional cooperativity between NPC1 and NPC2 in lipid transport. Proc Natl Acad Sci USA.

[52] Lu Albert, Tebar Francesc, Alvarez-Moya Blanca, López-Alcalá Cristina, Calvo Maria, Enrich Carlos, Agell Neus, Nakamura Takeshi, Matsuda Michiyuki, Bachs Oriol (2009). A clathrin-dependent pathway leads to KRas signaling on late endosomes en route to lysosomes. Journal of Cell Biology.

[53] Xu H, Ren D (2015). Lysosomal physiology. Annu Rev Physiol.

[54] Johnson DE, Ostrowski P, Jaumouille V, Grinstein S (2016). The position of lysosomes within the cell determines their luminal pH. J Cell Biol.

[55] Lawrence RE, Zoncu R (2019). The lysosome as a cellular centre for signalling, metabolism and quality control. Nat Cell Biol.

[56] Krug AW (2002). Human epidermal growth factor receptor-1 expression renders Chinese hamster ovary cells sensitive to alternative aldosterone signaling. J Biol Chem.

[57] Grant, B. D. & Donaldson, J. G. Pathways and mechanisms of endocytic recycling. *Nat Rev Mol Cell Biol* **10**, 597-608, 10.1038/nrm2755nrm2755 [pii] (2009). 10.1038/nrm2755 PMC3038567 19696797

[58] Takahashi S (2012). Rab11 regulates exocytosis of recycling vesicles at the plasma membrane. J Cell Sci.

[59] Prior IA (2001). GTP-dependent segregation of H-ras from lipid rafts is required for biological activity. Nat Cell Biol.

[60] Perera RM (2015). Transcriptional control of autophagy-lysosome function drives pancreatic cancer metabolism. Nature.

[61] Petersen Nikolaj H.T., Olsen Ole D., Groth-Pedersen Line, Ellegaard Anne-Marie, Bilgin Mesut, Redmer Susanne, Ostenfeld Marie S., Ulanet Danielle, Dovmark Tobias H., Lønborg Andreas, Vindeløv Signe D., Hanahan Douglas, Arenz Christoph, Ejsing Christer S., Kirkegaard Thomas, Rohde Mikkel, Nylandsted Jesper, Jäättelä Marja (2013). Transformation-Associated Changes in Sphingolipid Metabolism Sensitize Cells to Lysosomal Cell Death Induced by Inhibitors of Acid Sphingomyelinase. Cancer Cell.

[62] Eichner LJ (2019). Genetic Analysis Reveals AMPK Is Required to Support Tumor Growth in Murine Kras-Dependent Lung Cancer Models. Cell Metab.

[63] Kimmelman AC (2015). Metabolic Dependencies in RAS-Driven Cancers. Clin Cancer Res.

[64] Commisso C (2013). Macropinocytosis of protein is an amino acid supply route in Ras-transformed cells. Nature.

[65] Martinez-Lopez N, Athonvarangkul D, Mishall P, Sahu S, Singh R (2013). Autophagy proteins regulate ERK phosphorylation. Nat Commun.

[66] Schmick Malte, Vartak Nachiket, Papke Björn, Kovacevic Marija, Truxius Dina C., Rossmannek Lisaweta, Bastiaens Philippe I.H. (2014). KRas Localizes to the Plasma Membrane by Spatial Cycles of Solubilization, Trapping and Vesicular Transport. Cell.

[67] Chandra, A. *et al*. The GDI-like solubilizing factor PDEdelta sustains the spatial organization and signalling of Ras family proteins. *Nat Cell Biol* **14**, 148-158, 10.1038/ncb2394ncb2394 [pii] (2012). 10.1038/ncb2394 22179043

[68] Gagescu R (2000). The recycling endosome of Madin-Darby canine kidney cells is a mildly acidic compartment rich in raft components. Mol Biol Cell.

[69] Uchida Y., Hasegawa J., Chinnapen D., Inoue T., Okazaki S., Kato R., Wakatsuki S., Misaki R., Koike M., Uchiyama Y., Iemura S.-i., Natsume T., Kuwahara R., Nakagawa T., Nishikawa K., Mukai K., Miyoshi E., Taniguchi N., Sheff D., Lencer W. I., Taguchi T., Arai H. (2011). Intracellular phosphatidylserine is essential for retrograde membrane traffic through endosomes. Proceedings of the National Academy of Sciences.

[70] Finicle Brendan T., Ramirez Manuel U., Liu Gang, Selwan Elizabeth M., McCracken Alison N., Yu Jingwen, Joo Yoosun, Nguyen Jannett, Ou Kevin, Roy Saurabh Ghosh, Mendoza Victor D., Corrales Dania Virginia, Edinger Aimee L. (2018). Sphingolipids inhibit endosomal recycling of nutrient transporters by inactivating ARF6. Journal of Cell Science.

[71] Cho K.-j., Hill M. M., Chigurupati S., Du G., Parton R. G., Hancock J. F. (2011). Therapeutic Levels of the Hydroxmethylglutaryl-Coenzyme A Reductase Inhibitor Lovastatin Activate Ras Signaling via Phospholipase D2. Molecular and Cellular Biology.

[72] Gerl Mathias J., Sampaio Julio L., Urban Severino, Kalvodova Lucie, Verbavatz Jean-Marc, Binnington Beth, Lindemann Dirk, Lingwood Clifford A., Shevchenko Andrej, Schroeder Cornelia, Simons Kai (2012). Quantitative analysis of the lipidomes of the influenza virus envelope and MDCK cell apical membrane. The Journal of Cell Biology.

[73] Sampaio Julio L., Gerl Mathias J., Klose Christian, Ejsing Christer S., Beug Hartmut, Simons Kai, Shevchenko Andrej (2011). Membrane lipidome of an epithelial cell line. Proceedings of the National Academy of Sciences.

[74] Ma L, Ouyang Q, Werthmann GC, Thompson HM, Morrow EM (2017). Live-cell Microscopy and Fluorescence-based Measurement of Luminal pH in Intracellular Organelles. Front Cell Dev Biol.

[75] Zhou Y., Wong C.-O., Cho K.-j., van der Hoeven D., Liang H., Thakur D. P., Luo J., Babic M., Zinsmaier K. E., Zhu M. X., Hu H., Venkatachalam K., Hancock J. F. (2015). Membrane potential modulates plasma membrane phospholipid dynamics and K-Ras signaling. Science.

[76] Liang Hong, Mu Huanwen, Jean-Francois Frantz, Lakshman Bindu, Sarkar-Banerjee Suparna, Zhuang Yinyin, Zeng Yongpeng, Gao Weibo, Zaske Ana Maria, Nissley Dwight V, Gorfe Alemayehu A, Zhao Wenting, Zhou Yong (2019). Membrane curvature sensing of the lipid-anchored K-Ras small GTPase. Life Science Alliance.

[77] Ripley BD (1977). Modeling Spatial Patterns. J R Stat Soc Series B Stat Methodol.

[78] Diggle PJ, Mateu J, Clough HE (2000). A comparison between parametric and non-parametric approaches to the analysis of replicated spatial point patterns. Adv. in Appl. Probab..

