# Supplementary material for: Avicin G is a potent sphingomyelinase inhibitor and blocks oncogenic K- and H-Ras signaling
Source: Sci Rep. 2020 Jun 4;10:9120. doi: 10.1038/s41598-020-65882-5 (PMC7272413; doi:10.1038/s41598-020-65882-5)
Supplement: Supplementary file 1 — Supplementary Information. [file 41598_2020_65882_MOESM1_ESM.pdf]

Supplementary Data

Avicin G is a potent sphingomyelinase inhibitor and blocks oncogenic K- and H-Ras signaling.

Christian M. Garrido[1], Karen M. Henkels[1], Kristen M. Rehl[1], Hong Liang[2], Yong Zhou[2], Jordan Gutterman[3] and Kwang-jin Cho[1,*]

[1] Department of Biochemistry and Molecular Biology, School of Boonshoft Medical School, Wright State University, OH 45435

[2] Department of Integrative Biology and Pharmacology, McGovern Medical School, The University of Texas Health Science Center at Houston, TX 77030

[3] Department of Systems Biology, The University of Texas M. D. Anderson Cancer Center, Houston, TX 77030

* To whom correspondence should be addressed: Kwang-jin Cho, Department of Biochemistry and Molecular Biology, School of Boonshoft Medical School, Wright State University, Dayton, OH 45435; kwang-jin.cho@wright.edu; Tel: (937) 775-2670

Supplement Data 1

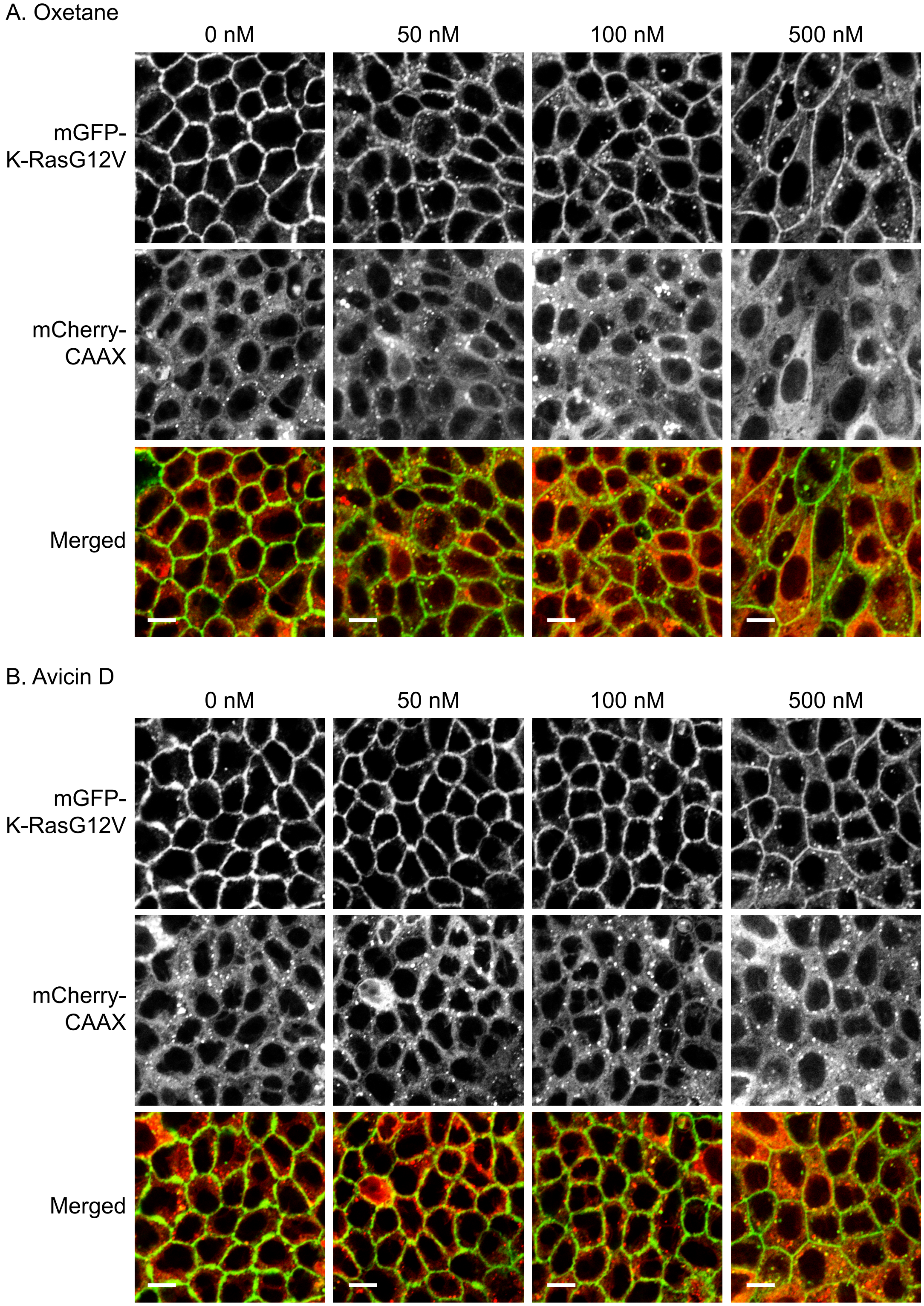

Figure S1. Avicin compounds mislocalize K-RasG12V from the PM. MDCK cells stably co-expressing mGFP-K-RasG12V and mCherry-CAAX were treated with (A) oxetane or (B) avicin D for 48h. Cells were fixed with 4% PFA and imaged in a confocal microscope. Scale bar 10 μm.

Supplement Data 2

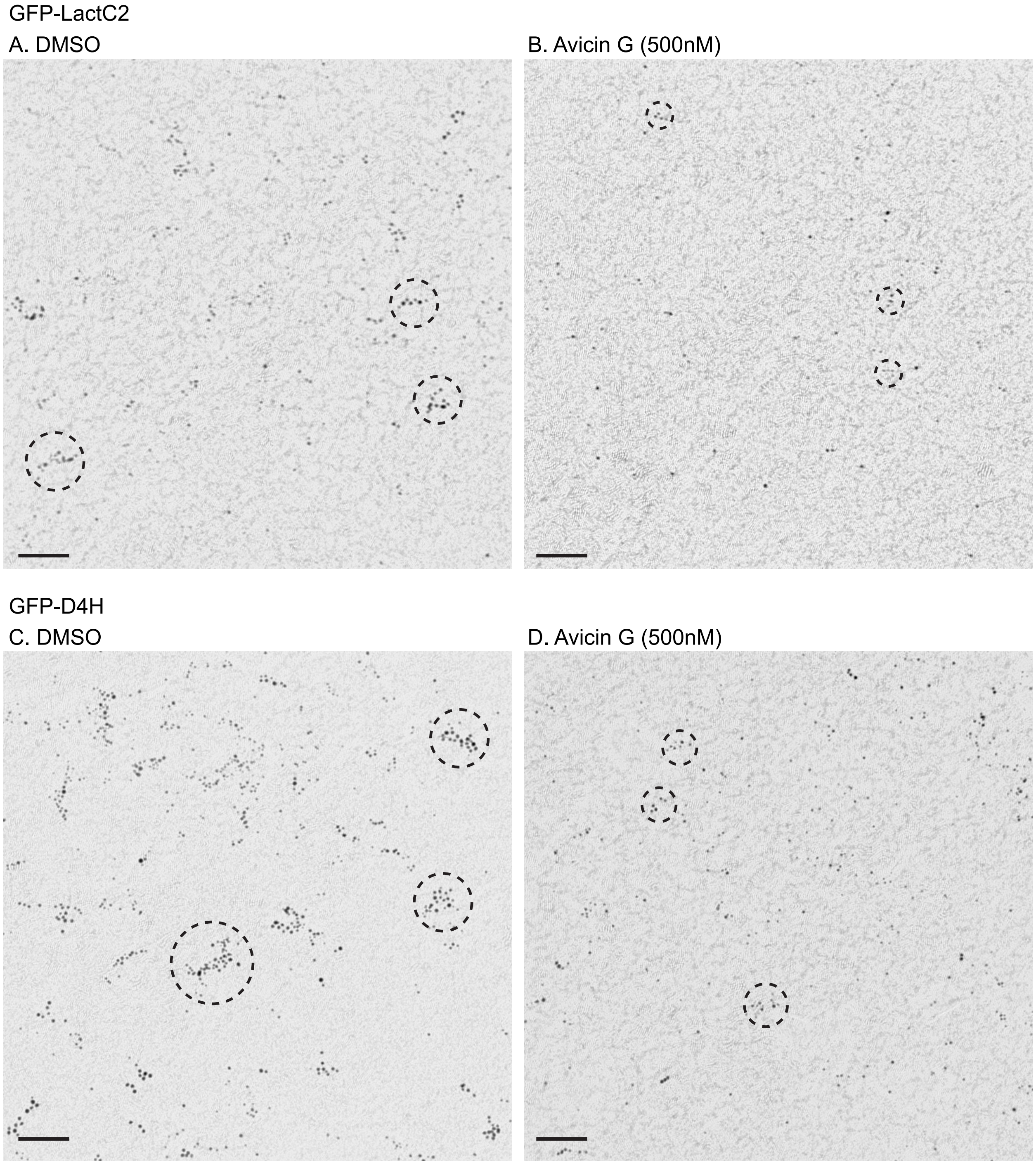

Figure S2. Avicin G disrupts the PM binding and nanoclustering of PtdSer and cholesterol at the PM. Intact apical PM sheets prepared from BHK cells expressing mGFP-LactC2 or -D4H and treated with 500nM avicin G for 48h were labeled with anti-GFP antibody directly coupled to 5nm gold and imaged in a transmission electron microscope (scale bar 100 nm). The nanoclusters of mGFP-LactC2 or -D4H at the PM are indicated by dotted circles. Representative micrographs are shown from n ≥15.

Suppement Data 3

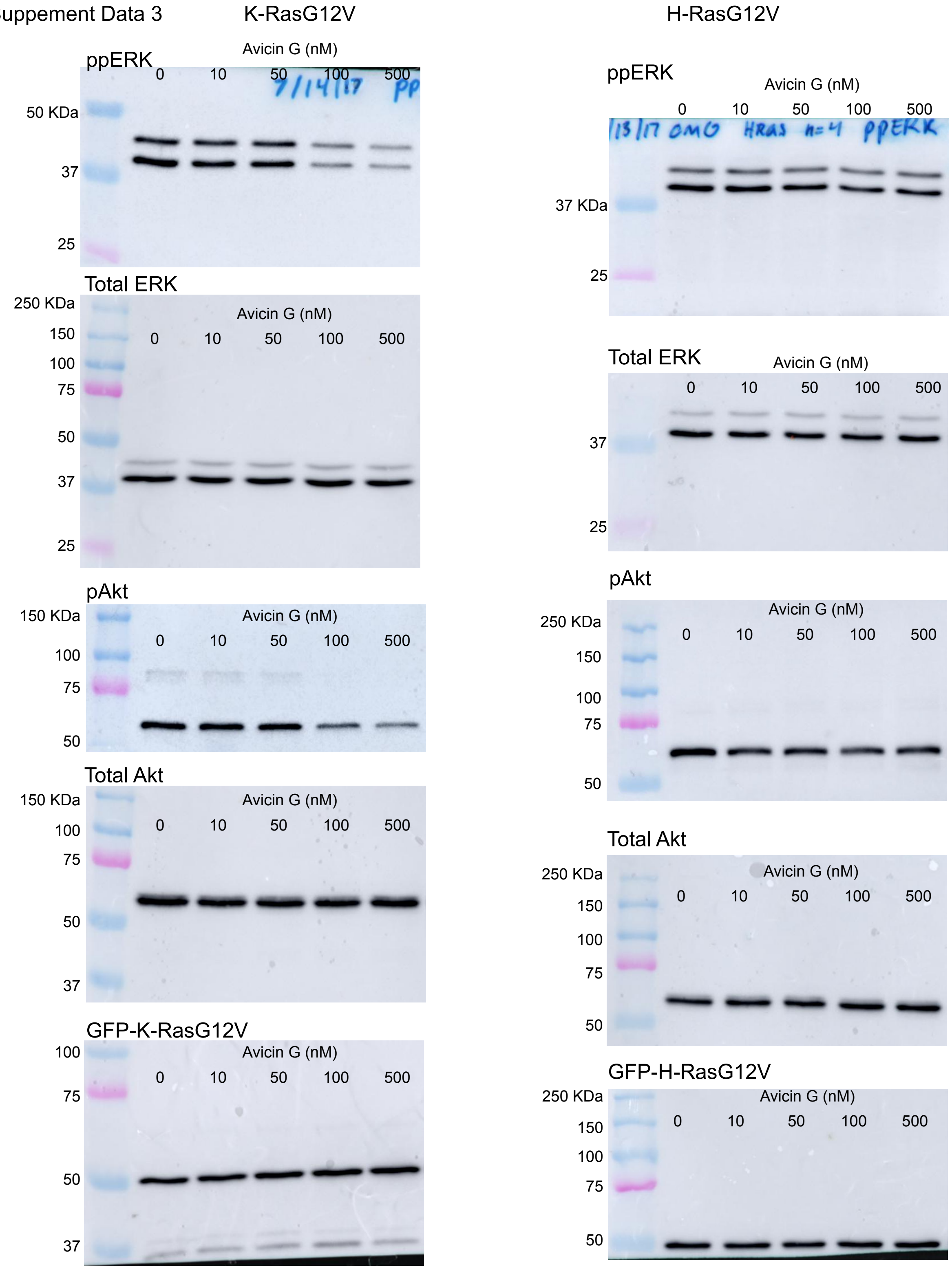

Figure S3. Avicin G blocks oncogenic Ras signal output. MDCK cells stably expressing mGFP-K-RasG12V or -H-RasG12V were treated with avicin G for 48h. Cell lysates were immunoblotted for phospho-ERK and -Akt (S473). Representative blots are shown from three independent experiments with total ERK and Akt blots being used as loading controls. An anti-GFP antibody was used to measure total mGFP-RasG12V levels.

Summelemnt Data 4

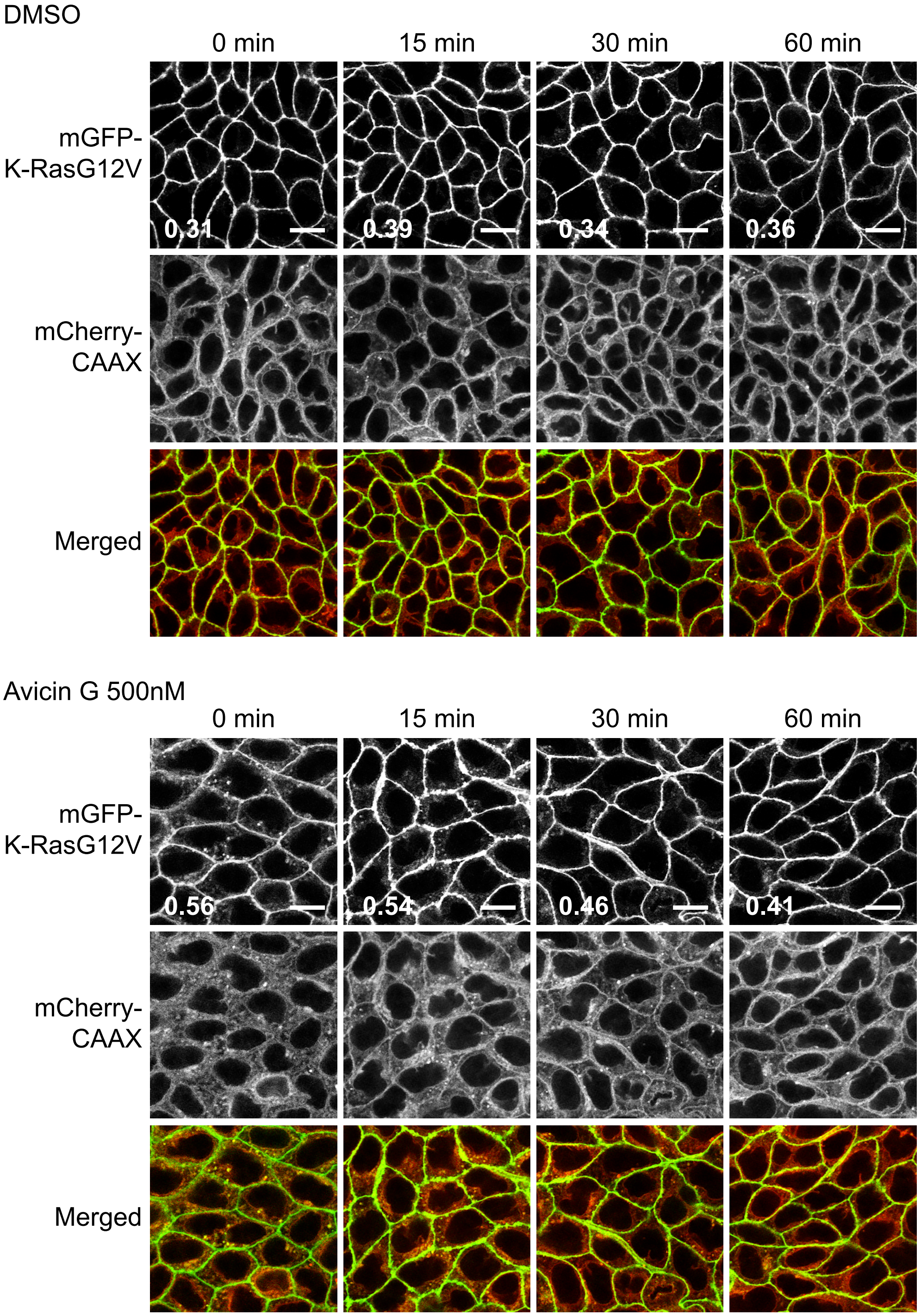

Figure S4. Exogenous PtdSer supplementation returns K-RasG12V to the PM. MDCK cells stably co-expressing mGFP-K-RasG12V and mCherry-CAAX were treated with 500nM avicin G for 48h. Cells were supplemented with 10μM exogenous PtdSer and incubated for the indicated time points. Cells were fixed with 4% PFA and imaged by confocal microscopy. Inserted values represent the mean of mCherry-CAAX co-localizing with mGFP-K-RasG12V calculated by Manders coefficient from three independent experiments. Scar bar = 10 μm.

Summelemnt Data 5

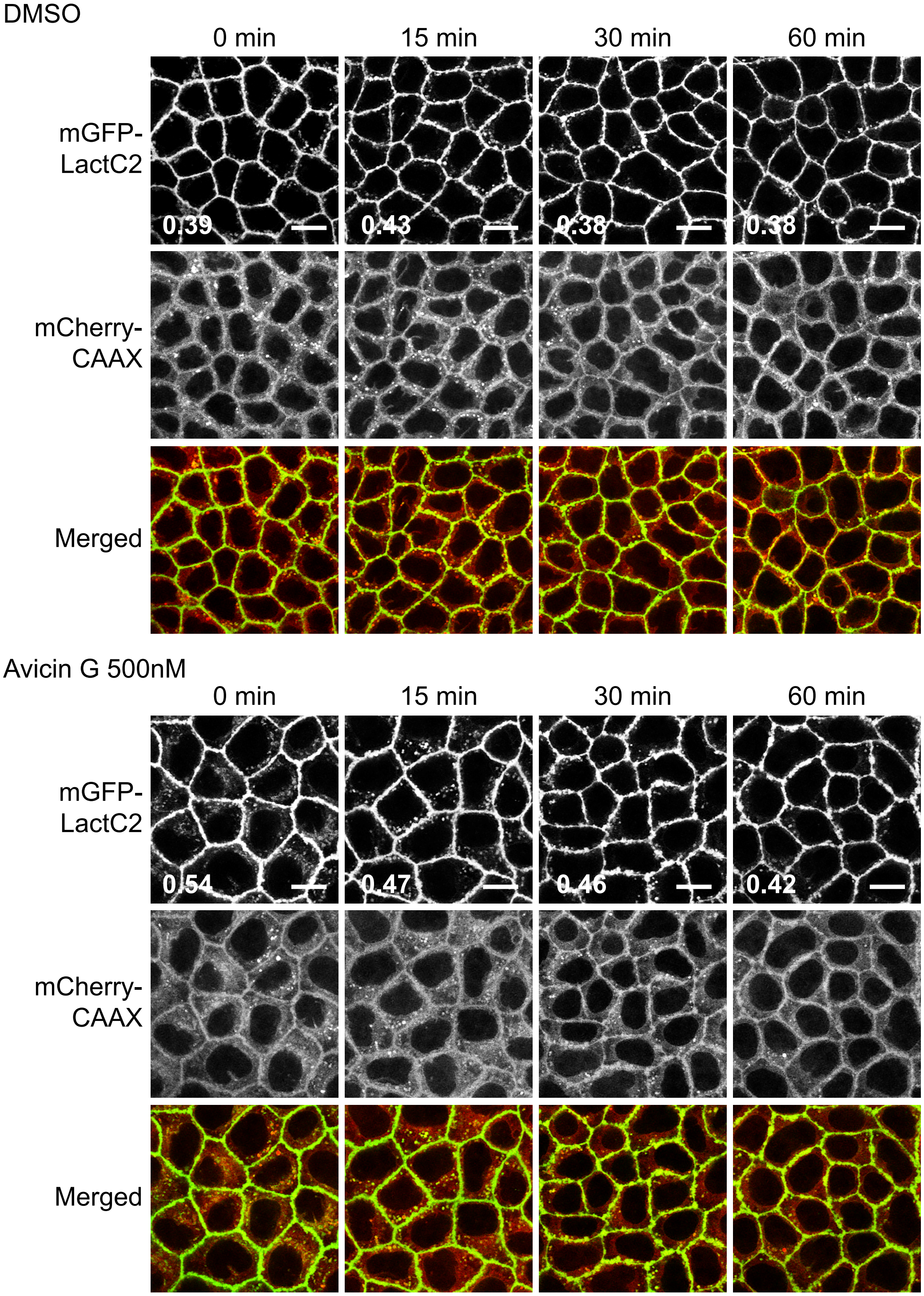

Figure S5. Exogenous PtdSer supplementation replenishes PtdSer at the PM. MDCK cells stably co-expressing mGFP-LactC2 and mCherry-CAAX were treated with 500nM avicin G for 48h. Cells were supplemented with 10μM exogenous PtdSer and incubated for the indicated time points. Cells were fixed with 4% PFA and imaged by confocal microscopy. Inserted values represent the mean of mCherry-CAAX co-localizing with mGFP-LactC2 calculated by Manders coefficient from three independent experiments. Scar bar = 10 μm.

Supplement Data 6

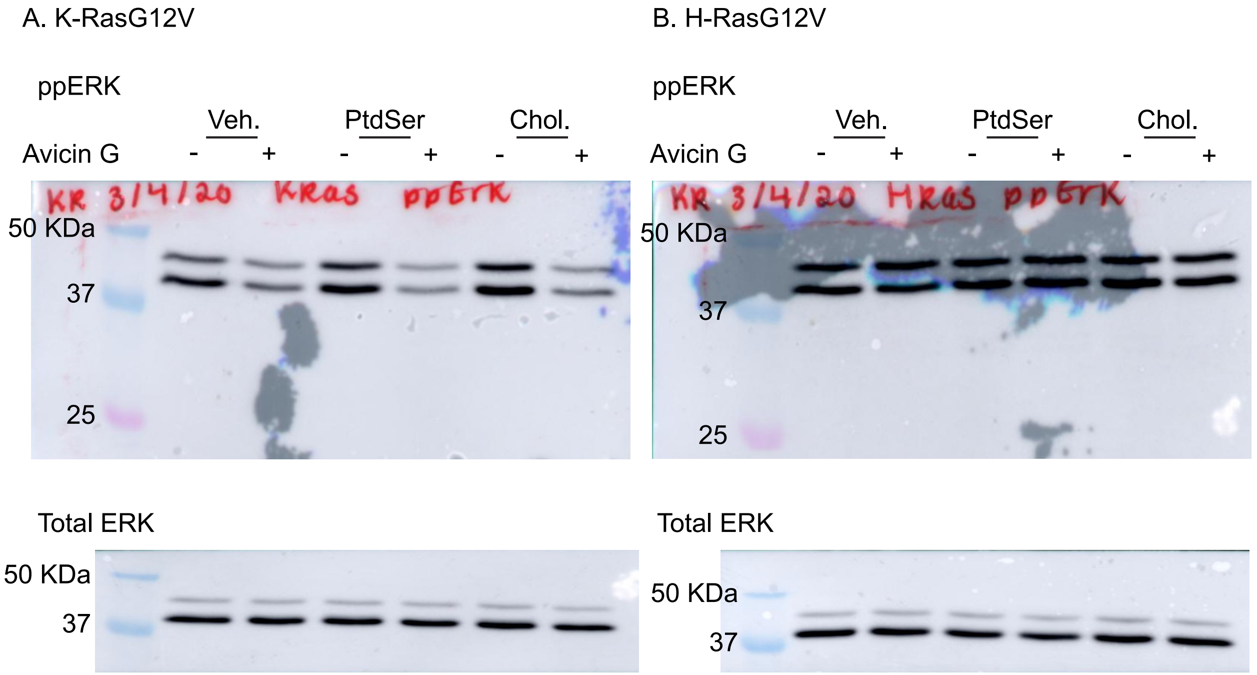

Figure S6. Exogenous cholesterol supplementation reactivates MAPK in H-Ras-transformed cells, but PtdSer supplementation does not reactivate MAPK in K-Ras-transformed cells. MDCK cells stably expressing mGFP-K-RasG12V (A) or -H-RasG12V (B) were treated with 500nM avicin G for 48h and incubated with 10μm exogenous PtdSer or cholesterol (Chol.) in the continued presence of 500nM avicin G for another 1h. Cell lysates were immunoblotted for phosphorylated ERK. Representative blots are shown from three independent experiments with total ERK blots being used as loading controls.

Supplement Data 7

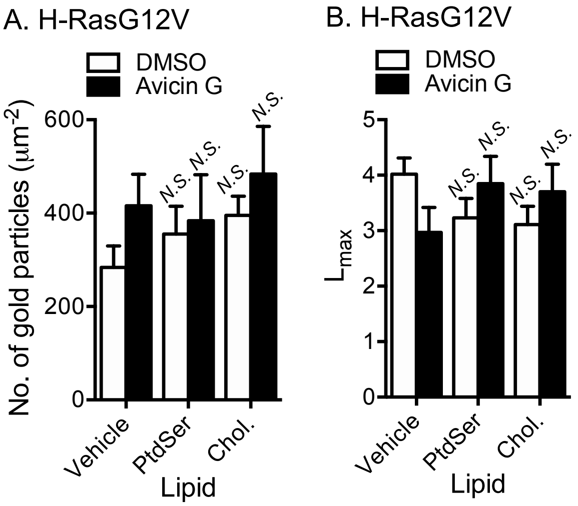

Figure S7. Supplementation of PtdSer or cholesterol does not change H-RasG12V PM binding and nanoclustering. (A) BHK cells expressing mGFP-H-RasG12V were treated with 500nM avicin G for 48h and incubated with 10μM exogenous PtdSer or cholesterol (Chol.) in the presence of 500nM avicin G for another 1h. Apical PM sheets were prepared and labeled with anti-GFP-conjugated gold and visualized EM. The graph shows a mean ± S.E.M (n ≥ 15). Significant differences between control (vehicle-treated) and lipid-supplemented cells were assessed by using a one-way ANOVA test (*N.S.* - not significant). (B) Spatial mapping of the same gold-labeled PM sheets was performed. The peak values, L_{max}, of the respective weighted mean K-function $L(r) - r$ curves are shown as bar graphs ($n \geq 15$). Significant differences between control (vehicle-treated) and lipid-supplemented cells were evaluated with bootstrap tests (*N.S.* – not significant).

Supplement Data 8

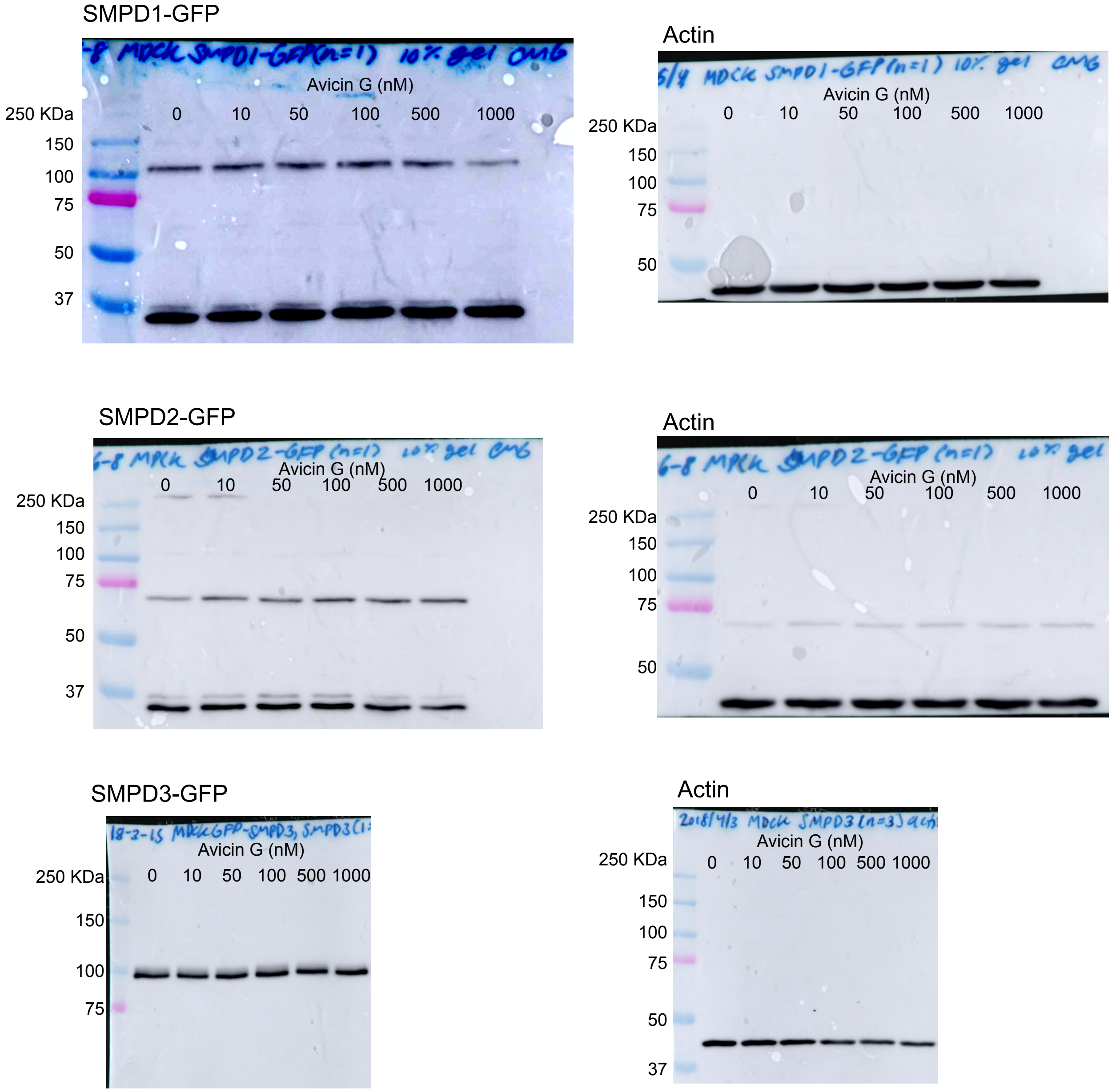

Figure S8. Avicin G perturbs SMases expression. MDCK cells stably expressing SMPD1-, SMPD2-, or SMPD3-GFP were treated with avicin G for 48h, and cell lysates were immunoblotted with an anti-GFP antibody to measure total SMPD-GFP expression levels. Representative blots are shown with actin blots being used as loading controls from three independent experiments.

Supplement Data 9

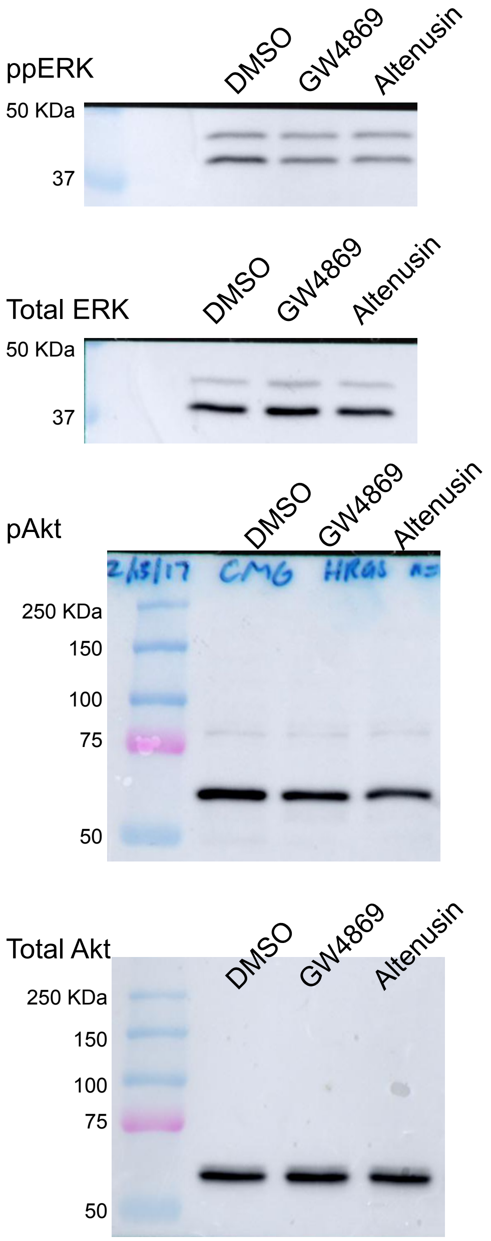

Figure S9. Neutral SMase inhibitors inhibits K-Ras signal output. Cell lysates of MDCK cells stably expressing mGFP-K-RasG12V treated with 20μM GW4869 or 20μM altenusin for 48h were immunoblotted for phospho-ERK and -Akt (S473). Representative blots are shown with total ERK and Akt blots as loading controls.
